# Supplementary material for: Small-molecule binding and sensing with a designed protein family
Source: Nat Commun. 2026 Mar 28;17:4533. doi: 10.1038/s41467-026-70953-8 (PMC13194776; doi:10.1038/s41467-026-70953-8)
Supplement: Supplementary file 1 — Supplementary Information [file 41467_2026_70953_MOESM1_ESM.pdf]

Supplementary Information for

Small-molecule binding and sensing with a designed protein family

Gyu Rie Lee[1,2,3,4‡], Samuel J. Pellock[1,2‡], Christoffer Norn[1,2‡], Doug Tischer[1,2], Justas Dauparas[1,2], Ivan Anishchenko[1,2], Jaron A. M. Mercer[5,6,7,8], Alex Kang[1,2], Asim K. Bera[1,2], Hannah Nguyen[1,2], Evans Brackenbrough[1,2], Banumathi Sankaran[9], Inna Goreshnik[1,2], Dionne Vafeados[1,2], Nicole Roullier[1,2], Hannah L. Han[1,2], Brian Coventry[1,2,3], Hugh K. Haddox[1,2], David R. Liu[5,6,7], Andy Hsien-Wei Yeh[10*], David Baker[1,2,3*]

[1]Department of Biochemistry, University of Washington, Seattle, WA, 98195, USA.
[2]Institute for Protein Design, University of Washington, Seattle, WA, 98195, USA.
[3]Howard Hughes Medical Institute, University of Washington, Seattle, WA, 98195, USA.
[4]Department of Biological Sciences, Korea Advanced Institute of Science and Technology, Daejeon 34141, Republic of Korea.
[5]Merkin Institute of Transformative Technologies in Healthcare, Broad Institute of Harvard and MIT, Cambridge, MA, 02142, USA.
[6]Department of Chemistry and Chemical Biology, Harvard University, Cambridge, MA, 02138, USA.
[7]Howard Hughes Medical Institute, Harvard University, Cambridge, MA, 02138, USA.
[8]Department of Chemistry and Biochemistry, University of California Santa Cruz, Santa Cruz, CA, 95064, USA.
[9]Berkeley Center for Structural Biology, Molecular Biophysics and Integrated Bioimaging, Lawrence Berkeley Laboratory, 1 Cyclotron Road, Berkeley, CA, 94720, USA.
[10]Department of Biomolecular Engineering, University of California Santa Cruz, Santa Cruz, CA, 95064, USA.

‡Equal contribution
*Co-corresponding authors. Email: dabaker@uw.edu (D.B.); hsyeh@ucsc.edu (A.H.W.Y.)

List of content:

Supplementary Fig. 1. NTF2 scaffold generation
Supplementary Fig. 2. Design metrics
Supplementary Fig. 3. FACS plots of yeast display library screening
Supplementary Fig. 4. Alphafold and Rosetta metrics of identified hits from yeast display and FACS
Supplementary Fig. 5. SEC elution profiles of nickel column eluates of putative binders identified from yeast display screening
Supplementary Fig. 6. Site-saturation mutagenesis of select binder hits from yeast display
Supplementary Fig. 7. Structure-guided redesign of roc22182 to disrupt dimerization interface
Supplementary Fig. 8. Biochemical and structural characterization of hcy129_mpnn5 and apx1049
Supplementary Fig. 9. Multidimensional scaling to visualize the structural similarity between NTF2-like designs and native KSI structures
Supplementary Fig. 10. Binding of each of the six representative binders measured against all six small-molecule targets
Supplementary Fig. 11. Optimization of the cortisol-binding protein hcy129
Supplementary Fig. 12. Library screening and hit characterization of designed CID minibinders

Supplementary Fig. 13. Characterization of hcy129.1_CID-miniH11 cortisol-induced dimerization
Supplementary Fig. 14. Cortisol sensor selectivity

Additional Supplementary Information:

- **Synthesis of biotin conjugated small-molecules**
- **Supplementary Fig. 15.** ITC binding isotherms
- **Supplementary Fig. 16.** FP binding isotherm for OHP-binding proteins
- **Supplementary Fig. 17.** FP binding isotherms for SN-38-binding proteins
- **Supplementary Fig. 18.** BLI titration sensograms for SN-38-binding proteins
- **Supplementary Fig. 19.** Binding traces of six representative binders against six small-molecule targets
- **Supplementary Fig. 20.** Size-exclusion chromatography profile of Bio-Rad protein standards
- **Supplementary Fig. 21.** Size-exclusion chromatography profiles of six representative binders used for target specificity assessment

Supplementary Table 1. Rosetta and AF2 metric cutoffs used to select designs
Supplementary Table 2. Small-molecule binding characterization results
Supplementary Table 3. Data collection and refinement statistics for the crystal structures of hcy129_mpnn5 and apx1049
Supplementary Table 4. The closest and farthest native KSI structure for each characterized binder measured by TMscore.
Supplementary Table 5. Chemical similarity between the targets and the ligands bound in KSI structures in the PDB represented by tanimoto coefficients
Supplementary Table 6. Binding affinities (K_D (μM)) of each of the six representative binders measured against all six targets

Supplementary References

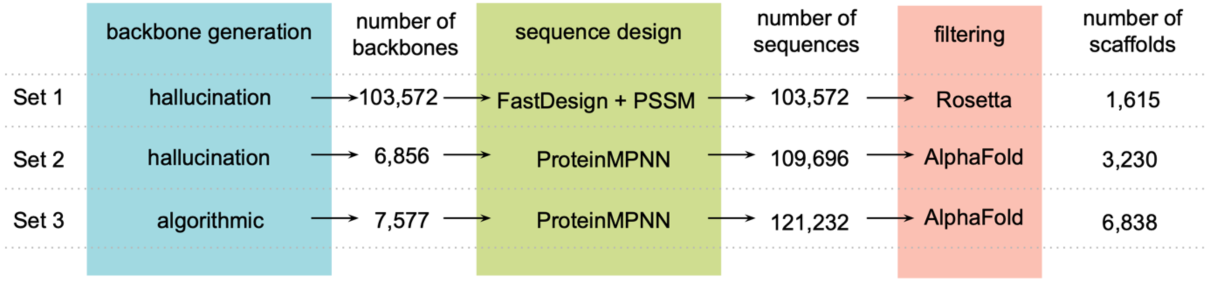

Supplementary Fig. 1. NTF2 scaffold generation. Computational pipelines to generate NTF2 scaffold sets.

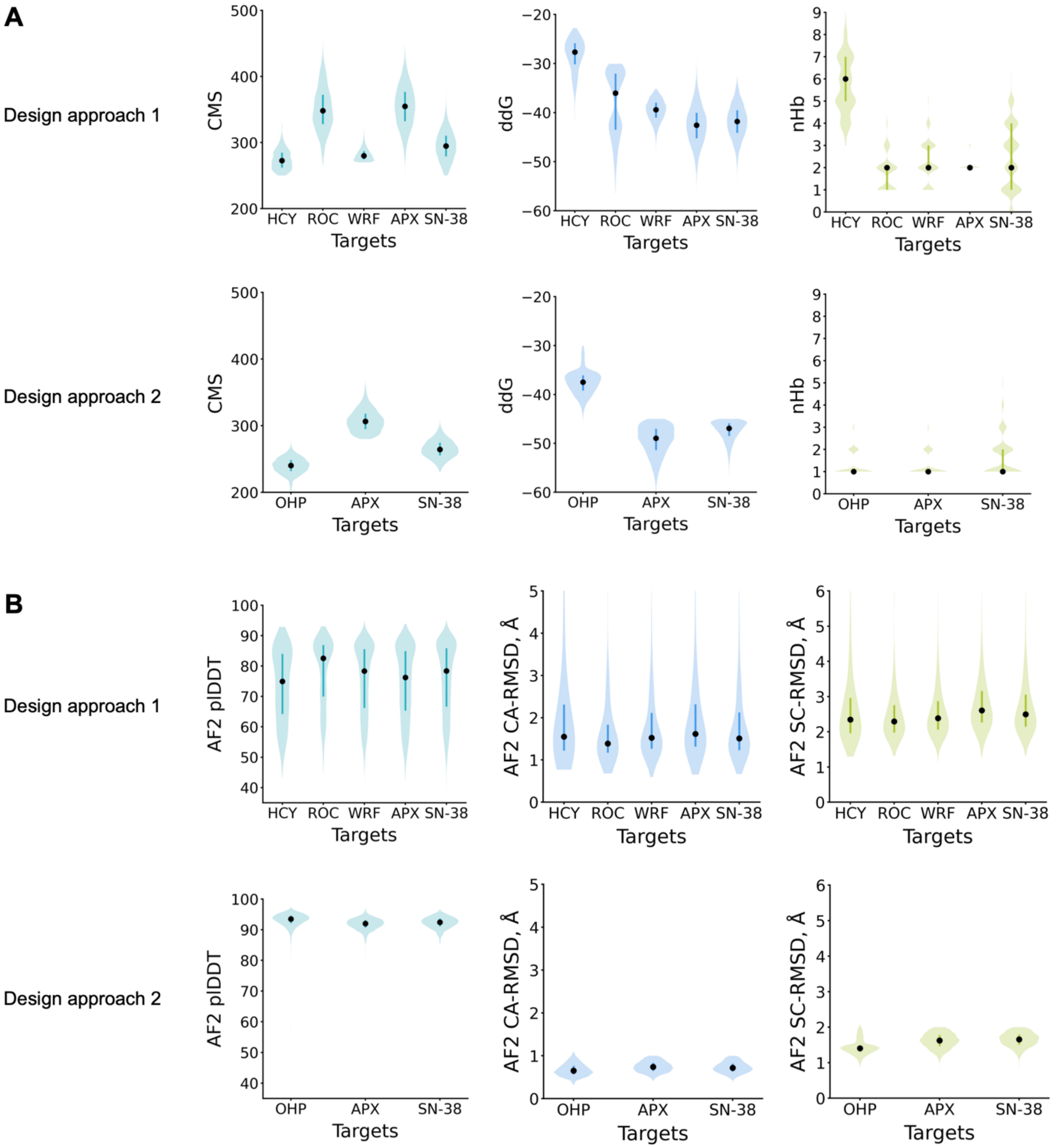

Supplementary Fig. 2. Design metrics. (**A**) Rosetta metrics of ordered designs for each ligand that were used for selection; contact molecular surface (CMS), ddG, and number of protein-ligand hydrogen bonds (nHb). (**B**) AlphaFold metrics of finally selected designs for each small-molecule. CA-RMSD and the binding site sidechain RMSD (SC-RMSD) values are shown for the subset of designs with plDDT higher than 80. For each violin plot the median (dot) and the first and third quartiles (line) are shown. Source data are provided as Source Data file.

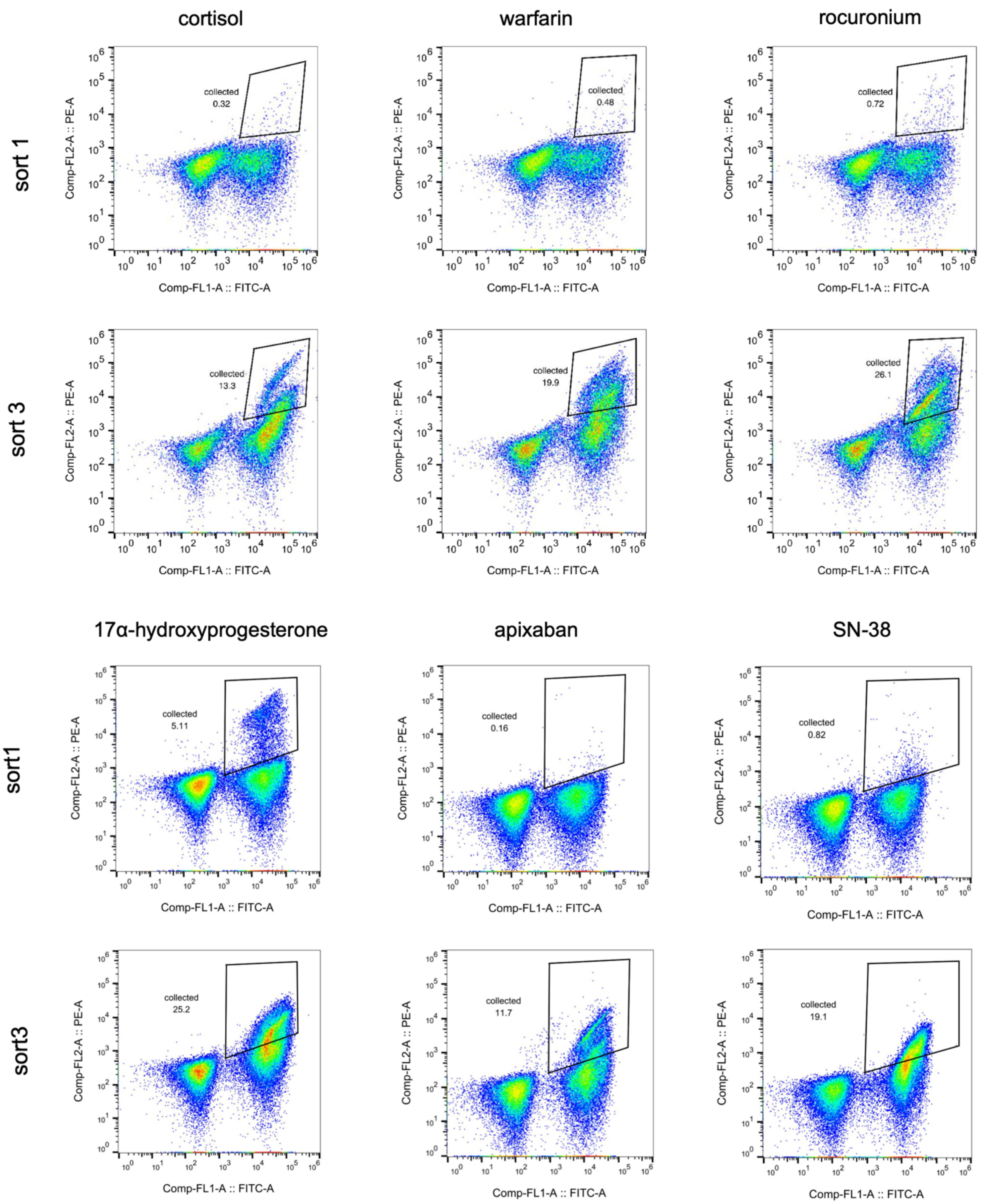

Supplementary Fig. 3. FACS plots of yeast display library screening. Representative FACS plots of designed protein yeast libraries. Libraries designed for cortisol, rocuronium, and warfarin were incubated with 1 μM of biotinylated small-molecule and 0.25 μM SAPE for all rounds. FACS results are shown for the 17α-hydroxyprogesterone (OHP) binder library incubated with 10uM biotin-OHP without avidity (round1) and 2nM without avidity (round3). For apixaban and SN-38 binder design libraries, FACS plots after incubation with 1uM biotinylated ligand with avidity (round1), and 100nM with avidity (round3) are shown.

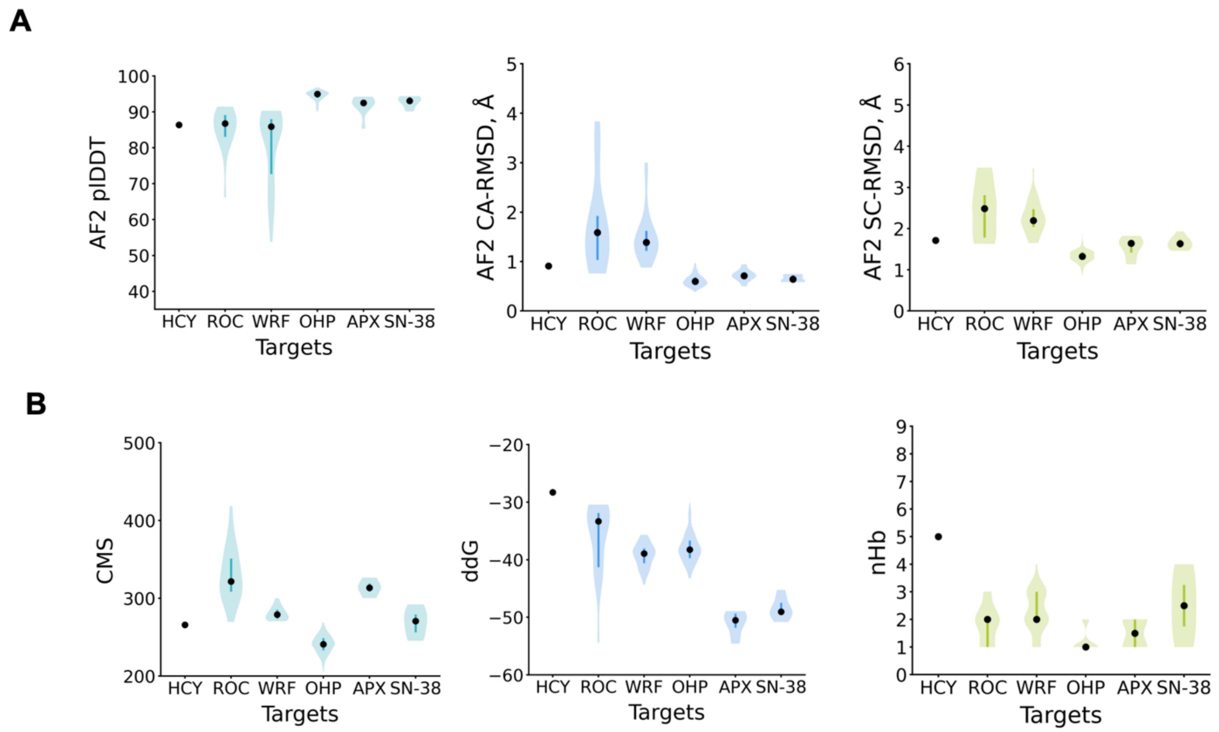

Supplementary Fig. 4. AlphaFold2 and Rosetta metrics of identified hits from yeast display and FACS. (A) AlphaFold2 (plDDT, Cα-RMSD of the models with plDDT > 80.0, and binding site side chain RMSD of the models with plDDT > 80.0) and **(B)** Rosetta metrics of putative binders identified by yeast display and FACS; contact molecular surface (CMS), ddG, and number of protein-ligand hydrogen bonds (nHb). For each violinplot the median (dot) and the first and third quartiles (line) are shown. Source data are provided as Source Data file.

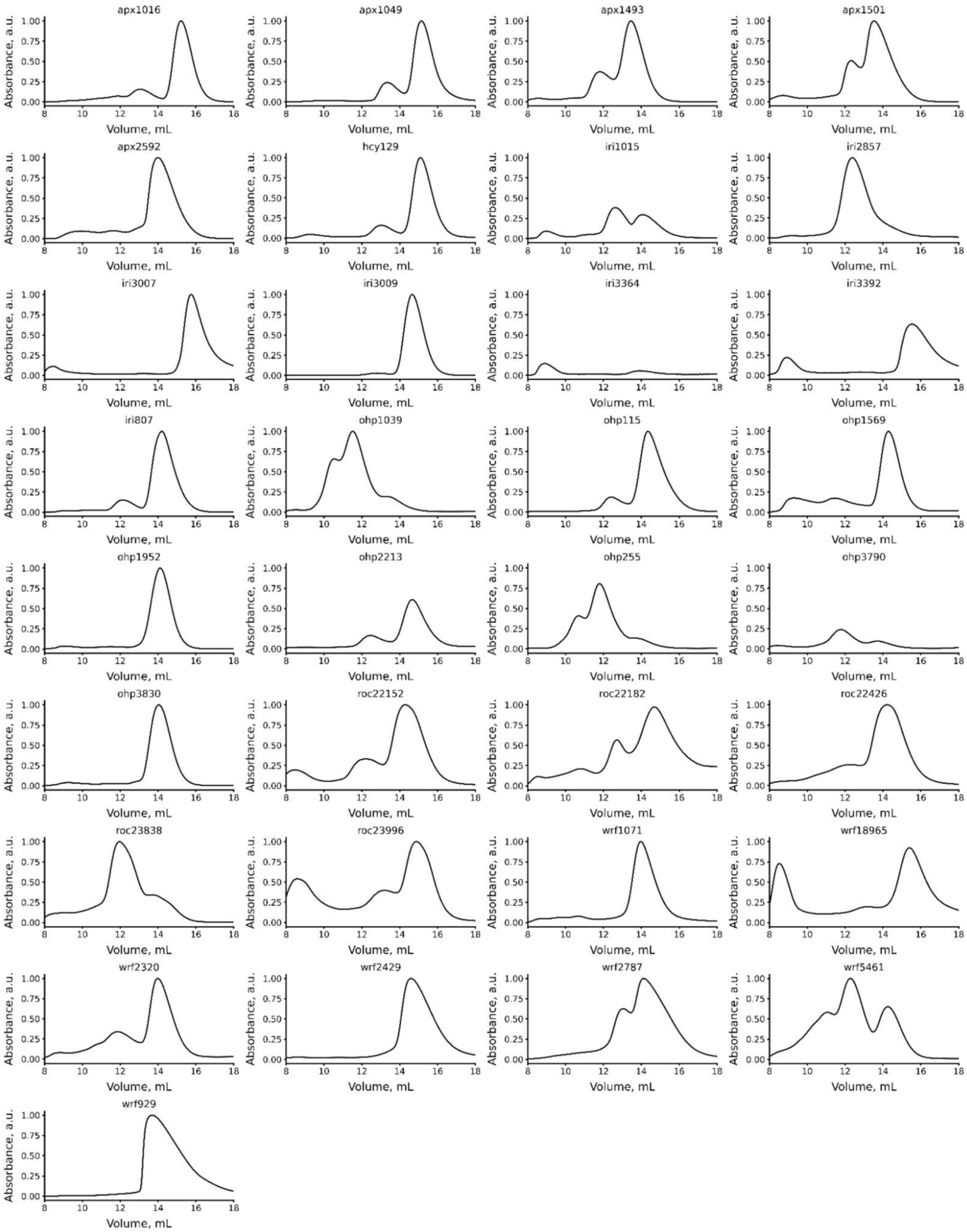

Supplementary Fig. 5. SEC elution profiles of nickel column eluates of putative binders identified from yeast display screening.

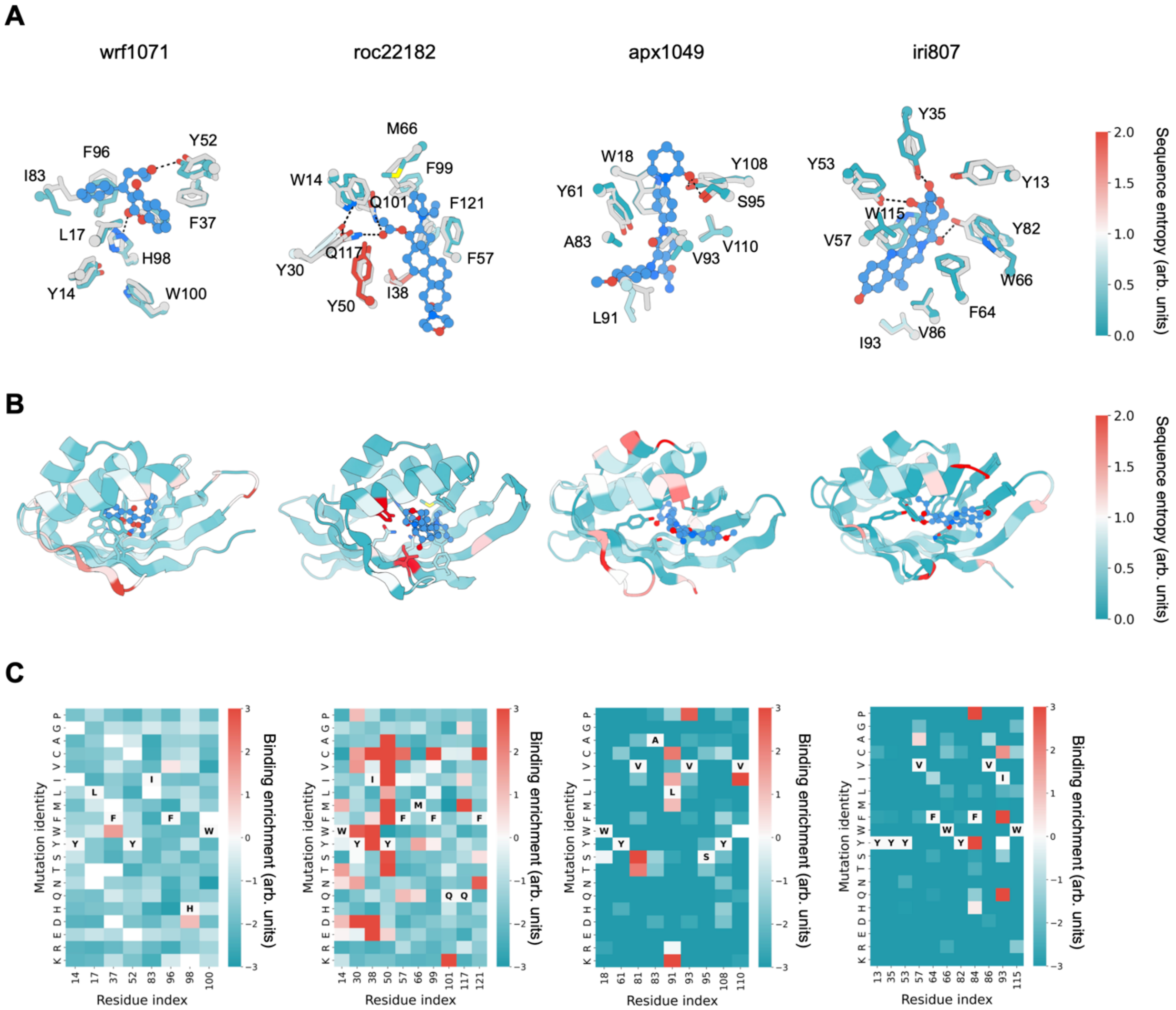

Supplementary Fig. 6. Site-saturation mutagenesis of select binder hits from yeast display. (**A**) Binding site overlays of design model (colored in heat map representing sequence entropy) and AF2 prediction (gray). (**B**) The design model colored by sequence entropy (color bar heatmap teal: conserved, red: high entropy) for each position. (**C**) Site-saturation mutagenesis enrichment plots for binding sites shown in panel **A** (teal: decreased binding, white: no change, and red: increased binding in reference to the wild type sequence). Sequence entropy and binding enrichment analysis was done for wrf1071 SSM sort2 with 1uM biotin-warfarin without avidity, roc22182 SSM sort2 with 2uM biotin-rocuronium without avidity, apx1049 SSM sort2 with 1nM biotin-apixaban without avidity, and iri807 SSM sort2 with 10nM biotin-SN-38 without avidity.

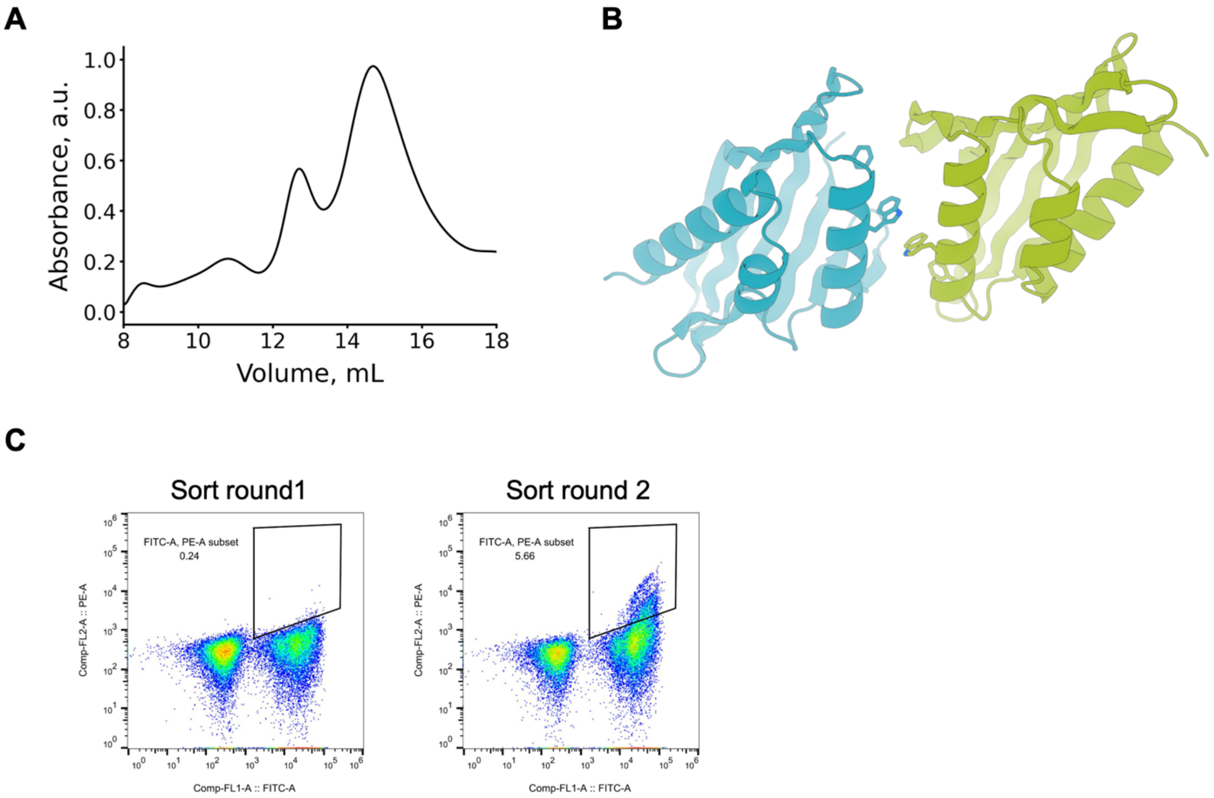

Supplementary Fig. 7. Structure-guided redesign of roc22182 to disrupt dimerization interface. (**A**) SEC trace of original roc22182 sequence identified from yeast display FACS screening. (**B**) Dimer interface of roc22182 predicted by AlphaFold-multimer. (**C**) FACS plots of roc22182 combinatorial library incubated at 1 μM and 100 nM biotin-rocuronium without avidity for consecutive two rounds of binding enrichment sorts.

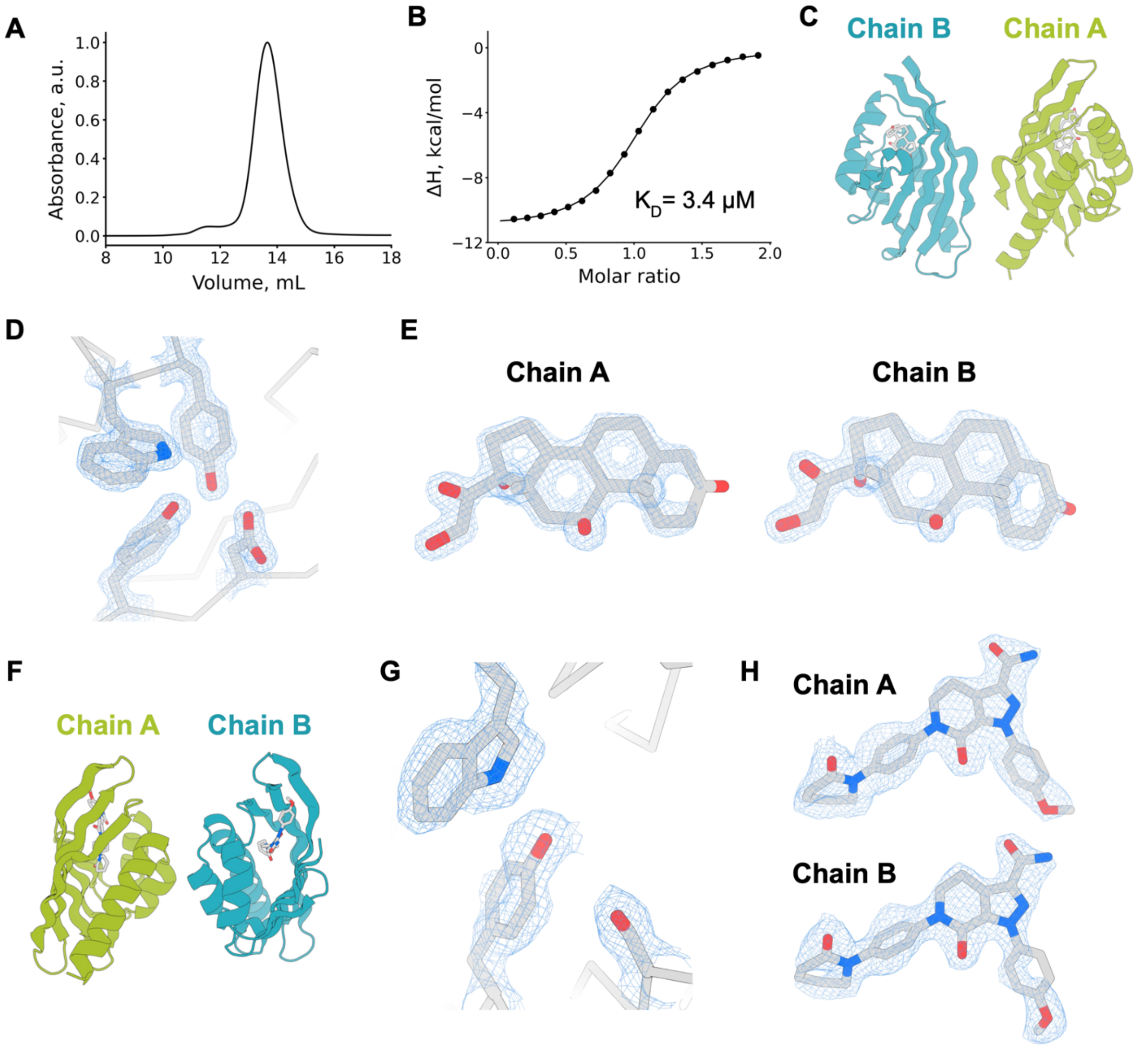

Supplementary Fig. 8. Biochemical and structural characterization of hcy129_mpnn5 and apx1049. (**A**) SEC of MPNN-redesigned hcy129 variant hcy129_mpnn5. (**B**) ITC binding isotherm and raw heat for hcy129_mpnn5 titrated with cortisol. Source data are provided as Source Data file. (**C**) Asymmetric unit of hcy129_mpnn5 crystal structure (chain A: teal; chain B: green). (**D**) Representative 2Fo-Fc electron density of protein side chains in the hcy129_mpnn5 crystal structure contoured at 2σ. (**E**) 2Fo-Fc electron density of the ligand in Chain A and Chain B of the crystal structure contoured at 2σ. (**F**) Asymmetric unit of apx1049 crystal structure (Chain A: green; Chain B: teal). (**G**) Representative 2Fo-Fc electron density of protein side chains in the apx1049 crystal structure contoured at 1 σ. (**H**) 2Fo-Fc electron density of the ligand in Chain A and Chain B contoured at 1 σ.

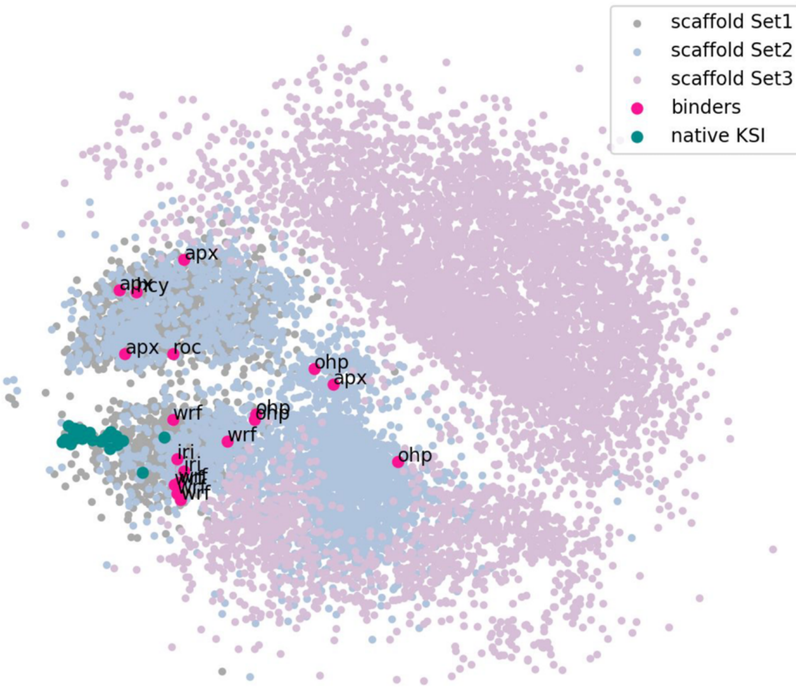

Supplementary Fig. 9. Multidimensional scaling to visualize the structural similarity between NTF2-like designs and native KSI structures. NTF2-like scaffolds generated using different approaches (scaffold sets 1, 2, and 3) are shown in light colors, characterized binders in magenta (labeled with target names), and ligand-bound Ketosteroid isomerase (KSI) structures in teal. Each dot represents one structure, and all pairwise distances for multidimensional scaling (MDS) were measured by (1.0 - TM-score).

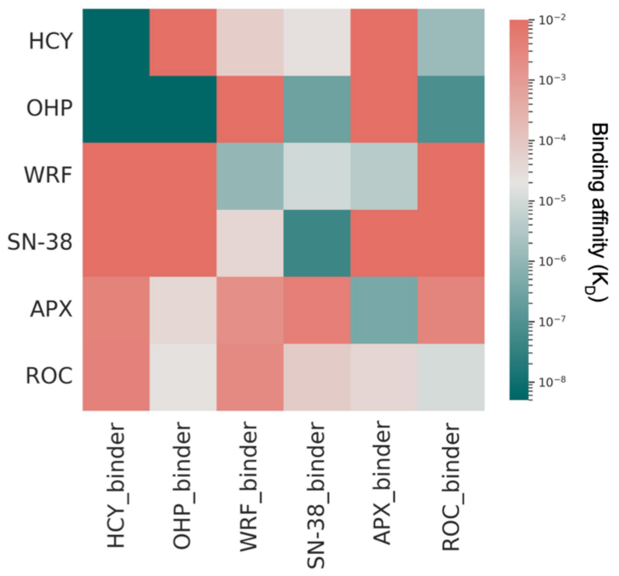

Supplementary Fig. 10. Binding of each of the six representative binders measured against all six small-molecule targets. Binding affinity K_D values measured for each of the six representative binders against all six small-molecule targets used in this study (**Supplementary Table 6**). The binders used for this experiment were hcy129.1, ohp1952, wrf1071, iri807.1, apx1049, and roc22182.1. Higher affinity is colored in green and lower in red (color bar unit in M). When no binding was observed, K_D of 10 mM (red) was assigned for visualization.

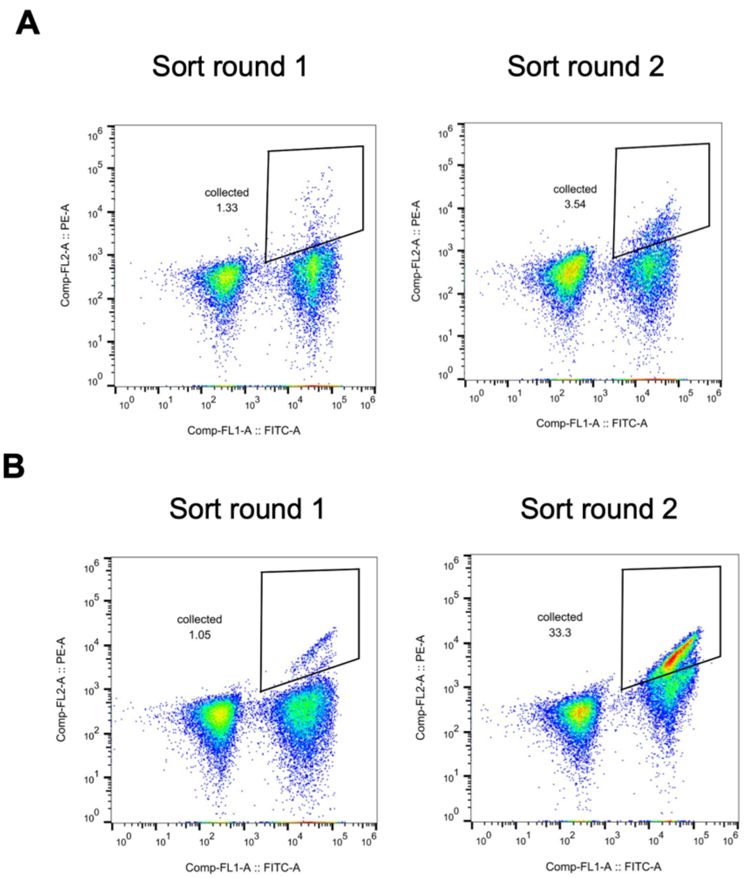

Supplementary Fig. 11. Optimization of the cortisol-binding protein hcy129. (**A**) FACS plots of yeast library of an SSM covering all positions of hcy129 with sort 1 at 1 μM with avidity (left), sort 2 at 100nM with avidity (right). (**B**) FACS plots of a hcy129 combinatorial mutant library containing favorable mutations identified from the SSM. Cells were collected for two consecutive rounds of FACS; for the first round the library was incubated with 100 pM biotin-cortisol without avidity, and the second round cell sorting was performed after incubation with 1 nM ligand without avidity.

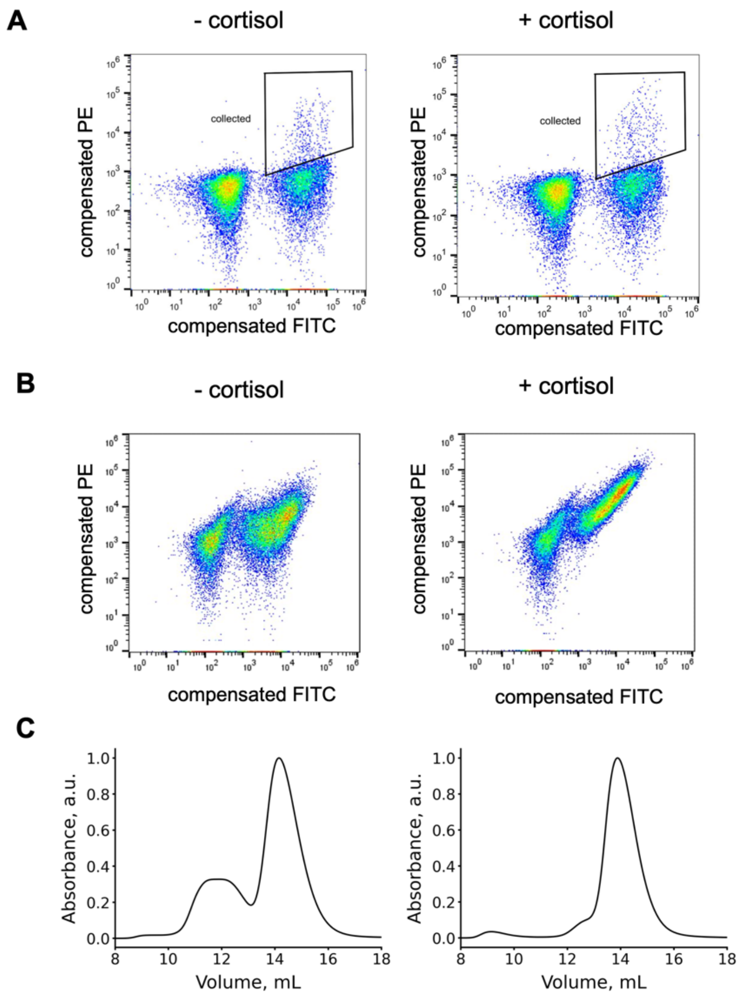

Supplementary Fig. 12. Library screening and hit characterization of designed CID minibinders. (**A**) FACS plots of yeast display library of minibinder CID library incubated with 1 μM biotinylated hcy129.1_CID in the absence (left) or presence (right) of 1 μM cortisol. (**B**) Representative single clone (miniH11) of yeast identified in libraries incubated with 0.2 μM biotinylated hcy129.1_CID in the presence (right) or absence (left) of 0.2 μM cortisol. (**C**) SEC traces of recombinant hcy129.1_CID (left) and miniH11 (right) proteins from *E. coli* expression.

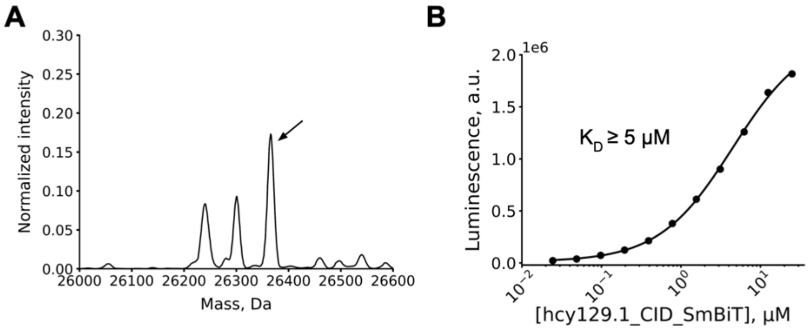

Supplementary Fig. 13. Characterization of hcy129.1_CID-miniH11 cortisol-induced dimerization. (**A**) Native mass spectrum of mixture of hcy129.1_CID (1 μM), miniH11 (1 μM), and cortisol (10 μM) shows a distinct peak (indicated by black arrow) for the mass of the ternary complex at 26366 Da (expected: 26359 Da). (**B**) Binding isotherm of miniH11-LgBiT (100 nM) titrated with hcy129.1_CID-SmBiT. Source data are provided as Source Data file.

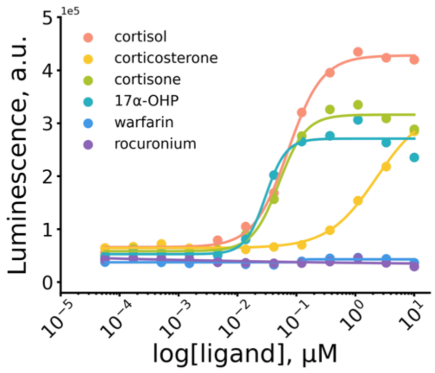

Supplementary Fig. 14. Cortisol sensor selectivity. A premixed solution of the cortisol binder hcy129.1_CID (200 nM) and minibinder designed for CID (miniH11, 200 nM) was titrated with various ligands and incubated for 2 hours, followed by addition of DTZ (25 µM) to initiate luminescence reaction. All reactions were carried out at 50 µL final volume in HBS-EP+ buffer (Cytiva). Source data are provided as Source Data file.

Additional Supplementary Information

Synthesis of biotin conjugated small-molecules

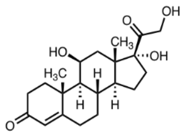

HCY

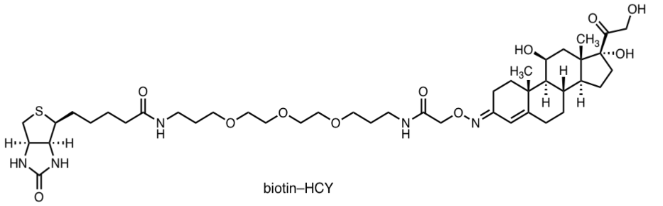

biotin–HCY

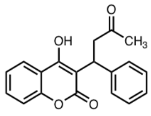

WRF

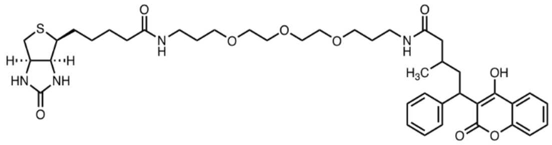

biotin–WRF

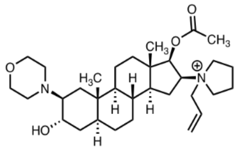

ROC

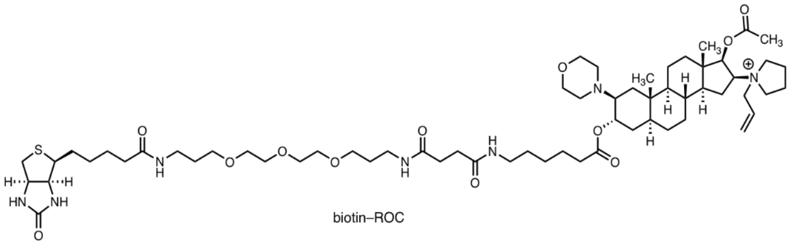

biotin–ROC

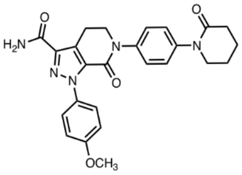

APX

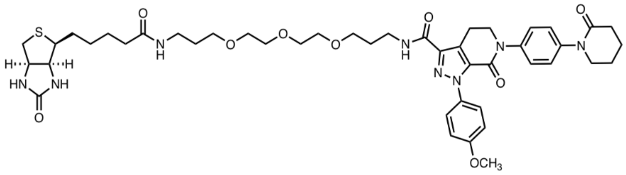

biotin–APX

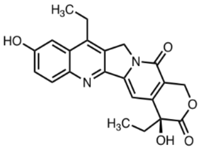

SN-38

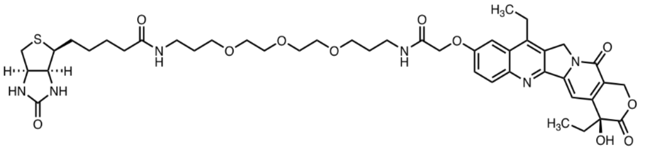

biotin–SN-38

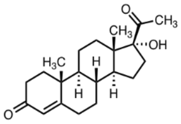

OHP

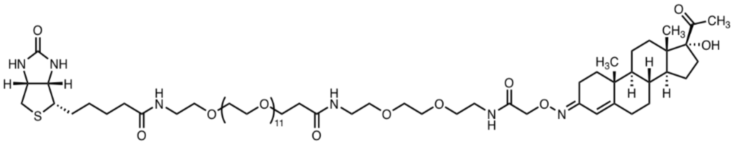

biotin–OHP

2D structure of the target small-molecule ligands and their biotin conjugates

Nuclear magnetic resonance

Proton nuclear magnetic resonance (^1H NMR) and carbon nuclear magnetic resonance (^{13}C NMR) spectra were recorded on a Bruker Ascend 400 spectrometer operating at 400 MHz for ^1H and 100 MHz for ^{13}C. Chemical shifts are reported in parts per million (ppm) with respect to residual protonated solvent for ^1H ($CHCl_3$ = δ 7.26) and with respect to carbon resonances of the solvent for ^{13}C (CDCl3 = δ 77.0). Peak multiplicities are annotated as follows: app = apparent, br = broad, s = singlet, d = doublet, t = triplet, q = quartet, p = quintet, m = multiplet.

Materials

Anhydrous solvents were purchased from Sigma-Aldrich. Hydrocortisone oxime (**S1**) was from Sigma-Aldrich. Biotin derivative **S2** was synthesized by previously reported methods[1] or purchased from MedChemExpress. Warfarin was from Oakwood. Rocuronium bromide was from MedChemExpress. Biotin derivative **S7** was previously described.[2] Apixaban derivative 1-(4-Methoxyphenyl)-7-oxo-6-(4-(2-oxopiperidin-1-yl)phenyl)-4,5,6,7-tetrahydro-1h-pyrazolo[3,4-c]pyridine-3-carboxylic acid (**S8**) was from Combi-Blocks. SN-38 derivative **S9** was previously described.[3] The biotin–OHP conjugate was previously described.[4]

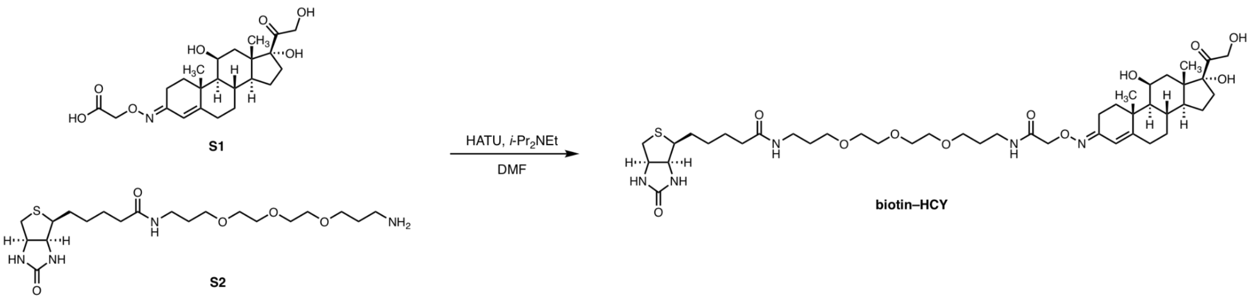

Biotin–HCY

N,N-Diisopropylethylamine (14.0 µL, 80.5 µmol, 3.5 equiv.) and HATU (11.4 mg, 29.9 µmol, 1.3 equiv.) were added sequentially to a solution of **S1** (10.0 mg, 23.0 µmol, 1 equiv.) and **S2** (10.2 mg, 23.0 µmol, 1 equiv.) in DMF (500 µL). After 1 hour, 1% aqueous TFA (300 µL) and methanol (100µL) were added. The mixture was purified by reverse-phase HPLC (Phenomenex C18 column, 5 µm particle size, 150 x 30 mm, 10 mL/min): 10% acetonitrile–water + 0.1% TFA, 10 min.; linear gradient 10→60% acetonitrile–water + 0.1% TFA, 40 min to afford the product biotin–HCY (10.0 mg, 50%).

HRMS (ESI) calcd. for $[C_{43}H_{69}N_5O_{11}S + H]^+$: 864.4788; found: 864.4878.

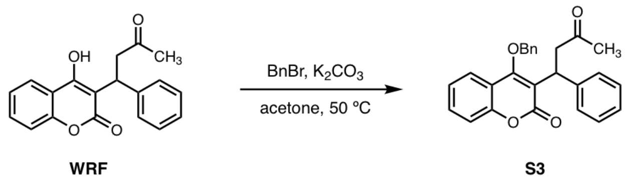

Benzyl warfarin **S3**

Potassium carbonate (269.0 mg, 1.946 mmol, 3.0 equiv.) and benzyl bromide (154.1 µL, 1.297 mmol, 2.0 equiv.) were added sequentially to a solution of warfarin (200 mg, 648.7 µmol, 1.0 equiv.) in acetone (0.2 M, 3.24 mL) at 0 ºC. The mixture was heated to 50 ºC. After 3 hours, the reaction mixture was cooled to 0 ºC and saturated aqueous ammonium chloride (1 mL) was added dropwise by pipet. The resulting mixture was transferred to a separatory funnel and partitioned between ethyl acetate (40 mL) and saturated aqueous ammonium chloride (40 mL). The aqueous portion was discarded and the organic portion was washed once with saturated aqueous sodium chloride (40 mL). The washed organics were dried with anhydrous sodium sulfate, filtered, and concentrated. The residue was purified by flash column chromatography (10→20% ethyl acetate/hexanes) to provide benzyl warfarin **S3** as a colorless semi-solid (110.0 mg, 43%).

R_f = 0.46 (20% ethyl acetate/hexanes)

^{1}H NMR (400 MHz, $CDCl_3$) δ 7.67 (dd, J = 8.0, 1.6 Hz, 1H), 7.55 (d, J = 8.5 Hz, 2H), 7.53 – 7.34 (m, 6H), 7.31 (d, J = 8.2 Hz, 1H), 7.29 – 7.20 (m, 2H), 7.21 – 7.15 (m, 1H), 7.18 – 7.00 (m, 1H), 5.33 (d, J = 11.4 Hz, 1H), 5.15 (d, J = 11.4 Hz, 1H), 5.03 (dd, J = 8.9, 6.1 Hz, 1H), 3.76 (dd, J = 18.2, 8.9 Hz, 1H), 3.25 (dd, J = 18.2, 6.1 Hz, 1H), 2.16 (s, 3H).

^{13}C NMR (101 MHz, $CDCl_3$) δ 207.46, 163.09, 162.42, 153.02, 141.95, 135.96, 131.46, 128.75, 128.53, 128.45, 127.87, 127.78, 126.70, 124.00, 123.60, 120.18, 117.03, 116.68, 75.89, 45.63, 36.46, 30.25.

MS (ESI) calcd. for $[C_{26}H_{22}O_4 + H]^+$: 399.16; found: 399.30

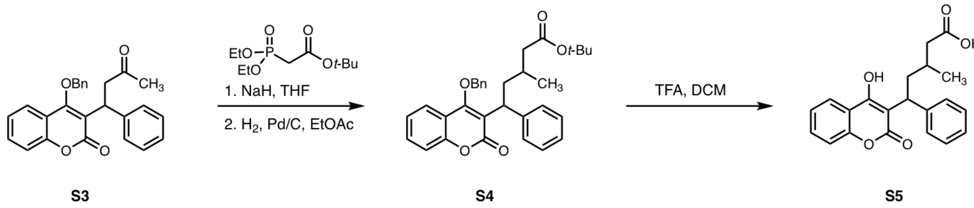

Warfarin acid **S5**

Sodium hydride (69.0 mg, 863.3 µmol, 8.0 equiv., 60% wt.) was added in two portions to a solution of *tert*-butyl diethylphosphonoacetate (101.4 µL, 431.6 µmol, 2.0 equiv.) and benzyl warfarin **S3** (86.0 mg, 215.8 µmol, 1.0 equiv.) in THF (0.1 M, 2.16 mL) at room temperature.

After 1 hour, excess sodium hydride was quenched by sequential addition of isopropanol (100 µL), methanol (100 µL), and saturated aqueous ammonium chloride (100 µL). The reaction mixture was transferred to a separatory funnel and partitioned between ethyl acetate (40 mL) and saturated aqueous sodium chloride (40 mL). The aqueous portion was discarded and the organic portion was washed once with saturated aqueous sodium chloride (40 mL). The washed organics were dried with anhydrous sodium sulfate, filtered, and concentrated to provide a crude yellow oil.

The crude residue was taken up in ethyl acetate (10 mL). Hydrogenation was effected in a ThalesNano H-Cube Mini Plus flow reactor by continuously cycling the reaction mixture through a Pd/C column (1 mL/min., 30 ºC) with 1 atmosphere of H_2. After 60 minutes, the reaction mixture was concentrated and the residue was purified by flash column chromatography (20→30% ethyl acetate/hexanes) to provide ester **S4** as a crisp colorless foam (12.7 mg, 14% for 2 steps).

R_f = 0.29 (30% ethyl acetate/hexanes)

Trifluoroacetic acid (600 µL) was added to a solution of S4 (22.0 mg, 53.8 µmol, 1.0 equiv.) in DCM (700 µL). After two hours, toluene (1 mL) was added and the reaction mixture was concentrated. The residue was purified by flash column chromatography (1→3% methanol/dichloromethane) to provide colorless solid **S5** as a 1.8:1 mixture of diastereomers (18.0 mg, 95%).

R_f = 0.15 (5% methanol/dichloromethane)

^{1}H NMR (400 MHz, $CDCl_3$, * indicates minor diastereomer) δ 7.77* (dd, J = 8.0, 1.6 Hz, 1H), 7.72 (dd, J = 8.0, 1.6 Hz, 1H), 7.56 – 7.45 (m, 3H), 7.44 – 7.18 (m, 5H), 4.69 (t, J = 7.7 Hz, 1H), 2.60 (dd, J = 15.7, 6.2 Hz, 1H), 2.43* (dd, J = 15.3, 6.6 Hz, 1H), 2.39 – 2.22 (m, 2H), 2.14 – 1.96 (m, 2H), 1.13* (d, J = 6.1 Hz, 3H), 1.07 (d, J = 6.4 Hz, 3H).

^{13}C NMR (101 MHz, $CDCl_3$, * indicates minor diastereomer) δ 178.60*, 178.50, 163.80*, 163.64, 160.43, 160.40*, 152.50, 141.55*, 140.87, 131.90, 131.83*, 129.52, 129.11*, 127.88, 127.79*, 127.62, 127.25*, 123.88, 123.85*, 122.85, 122.78*, 116.46*, 116.43, 116.03*, 116.01, 108.95, 108.57*, 41.36*, 41.32, 38.01*, 37.95, 37.77*, 37.52, 28.44*, 27.78, 20.32, 20.16*.

MS (ESI) calcd. for $[C_{21}H_{20}O_5 + H]^+$: 353.14; found: 353.23

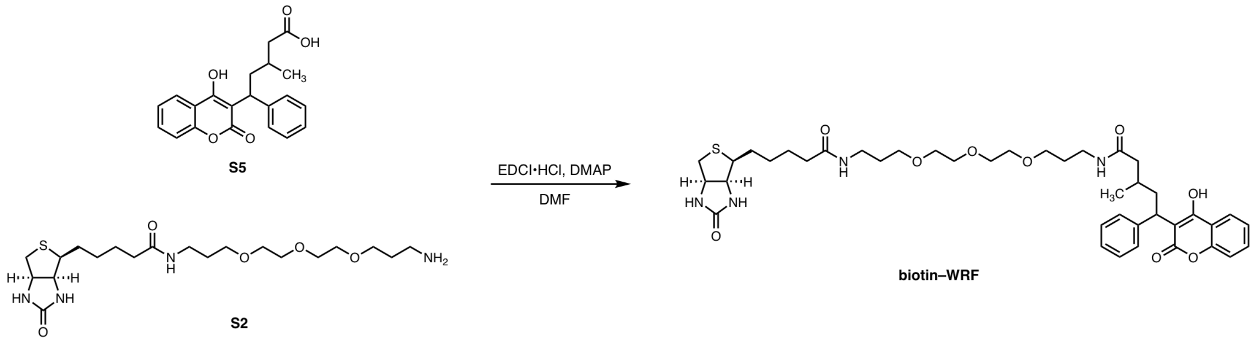

Biotin–WRF

DMAP (8.1 mg, 66.0 μmol, 4.0 equiv.) and EDCI•HCl (6.3 mg, 33.0 μmol, 1.3 equiv.) were added sequentially to a solution of **S5** (7.0 mg, 19.9 μmol, 1.2 equiv.) and **S2** (7.4 mg, 16.5 μmol, 1.0 equiv.) in DMF (500 μL). After 1 hour, 1% aqueous TFA (300 μL) and methanol (100μL) were added. The mixture was purified by reverse-phase HPLC (Phenomenex C18 column, 5 μm particle size, 150 x 30 mm, 10 mL/min): 10% acetonitrile–water + 0.1% TFA, 10 min.; linear gradient 10→60% acetonitrile–water + 0.1% TFA, 40 min to afford the product biotin–WRF (4.0 mg, 31%).

HRMS (ESI) calcd. for $[C_{41}H_{56}N_4O_9S + H]^+$: 781.3841; found: 781.3725.

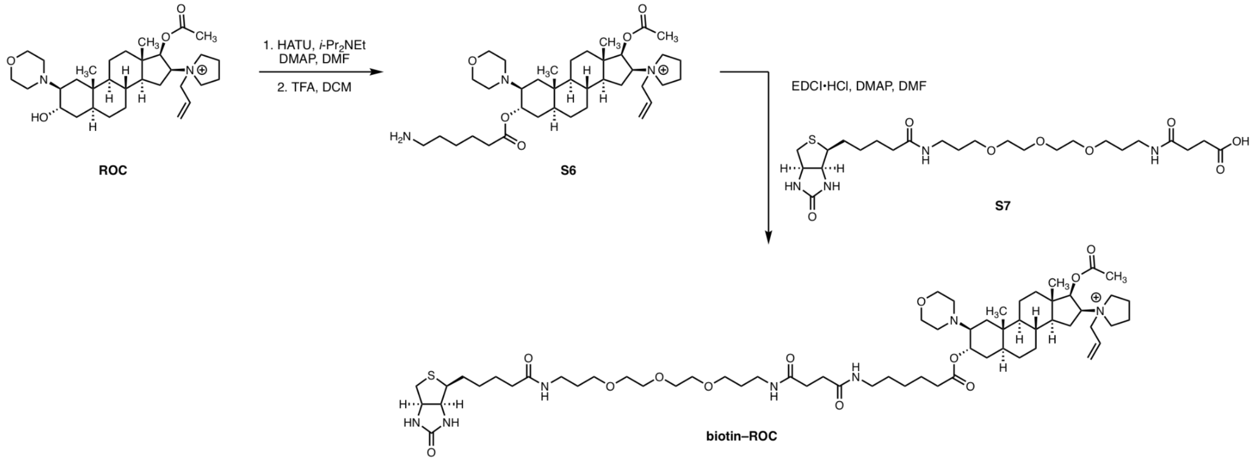

Biotin–ROC

DMAP (36.1 mg, 295.2 μmol, 6.0 equiv.), HOBt hydrate (7.5 mg, 49.2 μmol, 1.0 equiv.), and EDCI•HCl (28.3 mg, 147.6 μmol, 3.0 equiv.) were added sequentially to a solution of rocuronium bromide (30.0 mg, 49.2 μmol, 1.0 equiv.) and *N*-Boc-6-aminohexanoic acid (34.1 mg, 147.6 μmol, 3.0 equiv.) in DMF (500 μL). Reaction progress was monitored by LCMS. After 2 hours the reaction mixture was concentrated by rotary evaporation and the resulting residue was taken up in DCM (500 μL). The mixture was cooled to 0 ºC and TFA (300 μL) was added. The mixture was allowed to warm to room temperature. After two hours, toluene (500 μL) was added and the reaction mixture was concentrated by rotary evaporation to provide a crude mixture containing **S6** that was used in the next step without purification.

DMAP (4.5 mg, 36.5 µmol, 5.0 equiv.) and EDCI•HCl (4.2 mg, 21.9 µmol, 3.0 equiv.) were added sequentially to a solution of crude **S6** (up to 49.2 µmol, 6.7 equiv.) and **S7** (4.0 mg, 7.3 µmol, 1.0 equiv.) in DMF (500 µL). After 1 hour, 1% aqueous TFA (300 µL) and methanol (100µL) were added. The mixture was purified by reverse-phase HPLC (Phenomenex C18 column, 5 µm particle size, 150 x 30 mm, 10 mL/min): linear gradient 10→90% acetonitrile–water + 0.1% TFA, 60 min to afford the product biotin–ROC (4.0 mg, 31%).

HRMS (ESI) calcd for $[C_{62}H_{104}N_7O_{12}S]^+$: 1170.7459; found: 1170.7530.

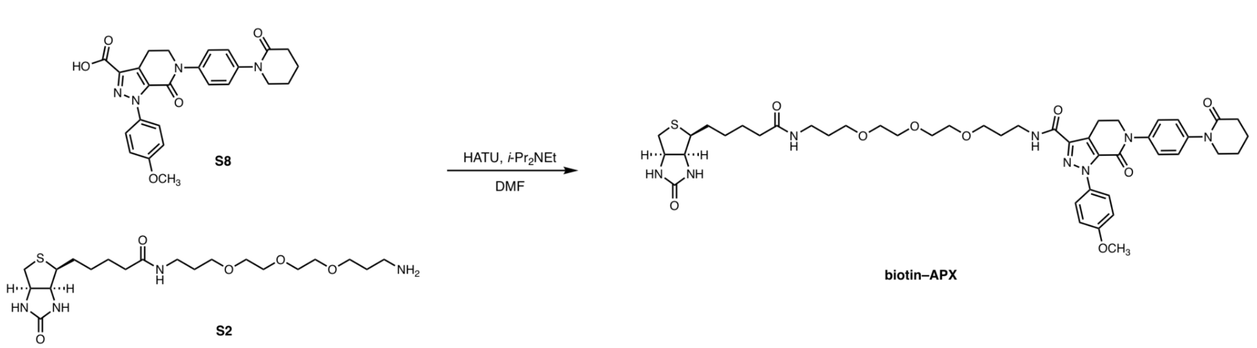

Biotin–APX

N,N-Diisopropylethylamine (15.5 µL, 89.3 µmol, 1.5 equiv.) and HATU (17.6 mg, 46.4 µmol, 1.3 equiv.) were added sequentially to a solution of **S8** (19.7 mg, 42.8 µmol, 1.2 equiv.) and **S2** (15.9 mg, 35.7 µmol, 1 equiv.) in DMF (500 µL). After 1 hour, 1% aqueous TFA (300 µL) and methanol (100µL) were added. The mixture was purified by reverse-phase HPLC (Phenomenex C18 column, 5 µm particle size, 150 x 30 mm, 10 mL/min): 10% acetonitrile–water + 0.1% TFA, 10 min.; linear gradient 10→60% acetonitrile–water + 0.1% TFA, 40 min to afford the product biotin–APX (15.0 mg, 47%).

HRMS (ESI) calcd for $[C_{45}H_{60}N_8O_9S + H]^+$: 889.4277; found: 889.4346.

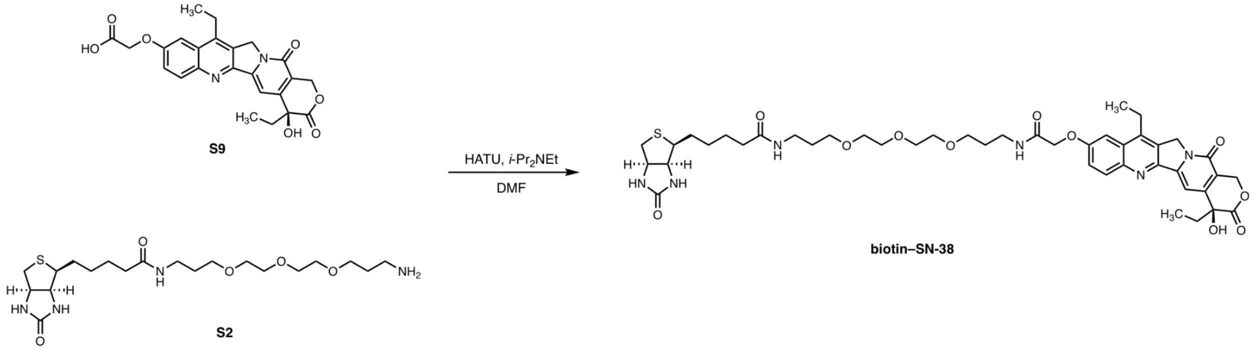

Biotin–SN-38

N,N-Diisopropylethylamine (26.7 μL, 153.3 μmol, 3.0 equiv.) and HATU (38.8 mg, μmol, 2.0 equiv.) were added sequentially to a solution of **S9** (23.0 mg, 51.1 μmol, 1.0 equiv.) and **S2** (34.2 mg, 76.6 μmol, 1.5 equiv.) in DMF (500 μL). After 1 hour, 1% aqueous TFA (300 μL) and methanol (100μL) were added. The mixture was purified by reverse-phase HPLC (Phenomenex C18 column, 5 μm particle size, 150 x 30 mm, 10 mL/min): 10% acetonitrile–water + 0.1% TFA, 10 min.; linear gradient 10→60% acetonitrile–water + 0.1% TFA, 40 min to afford the product biotin–SN-38 (18.0 mg, 40%).

HRMS (ESI) calcd for $[C_{44}H_{58}N_6O_{11}S + H]^+$: 879.3958; found: 879.4061.

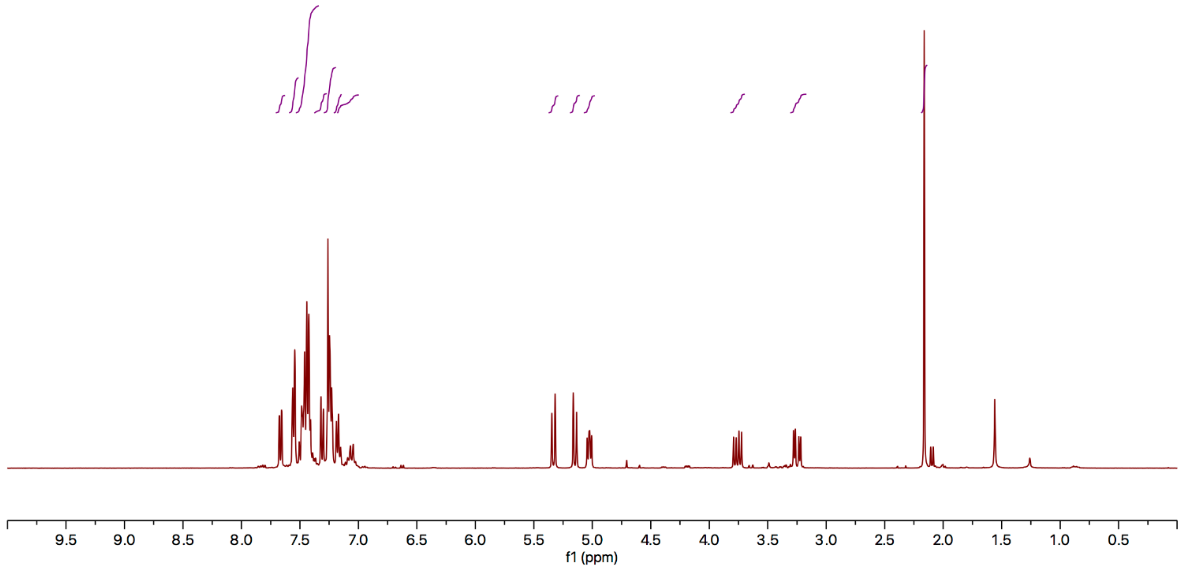

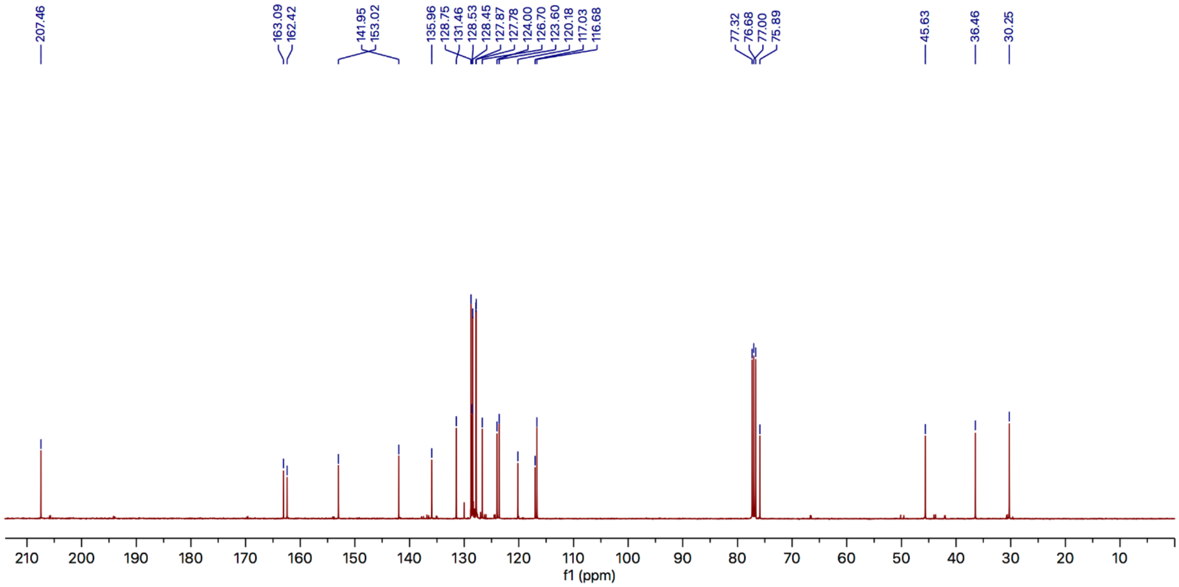

^{1}H (top) and ^{13}C (bottom) NMR spectra of compound **S3**

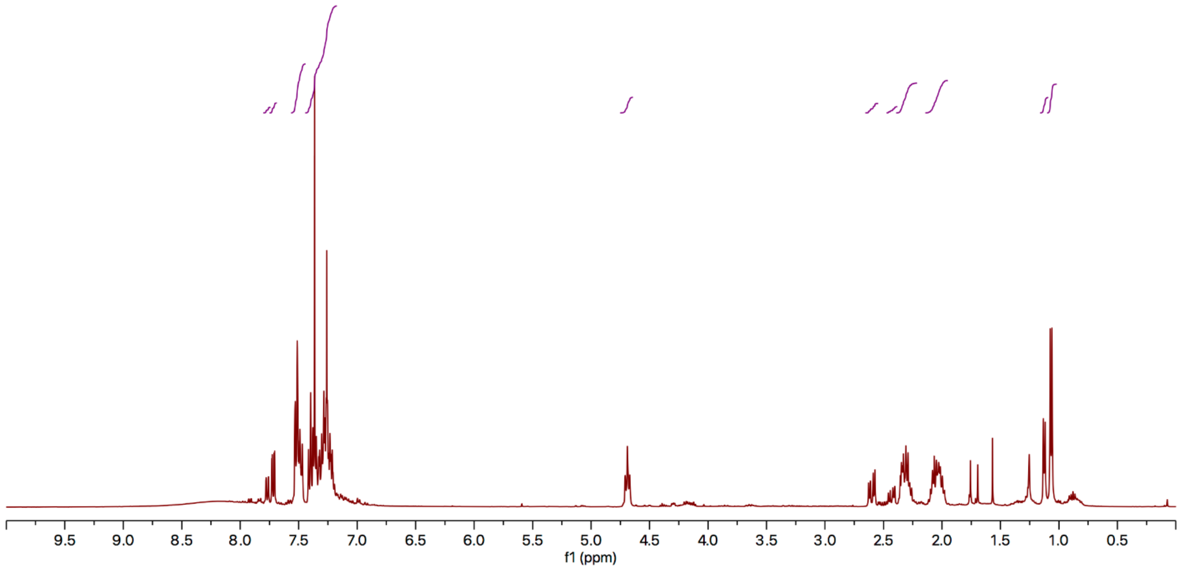

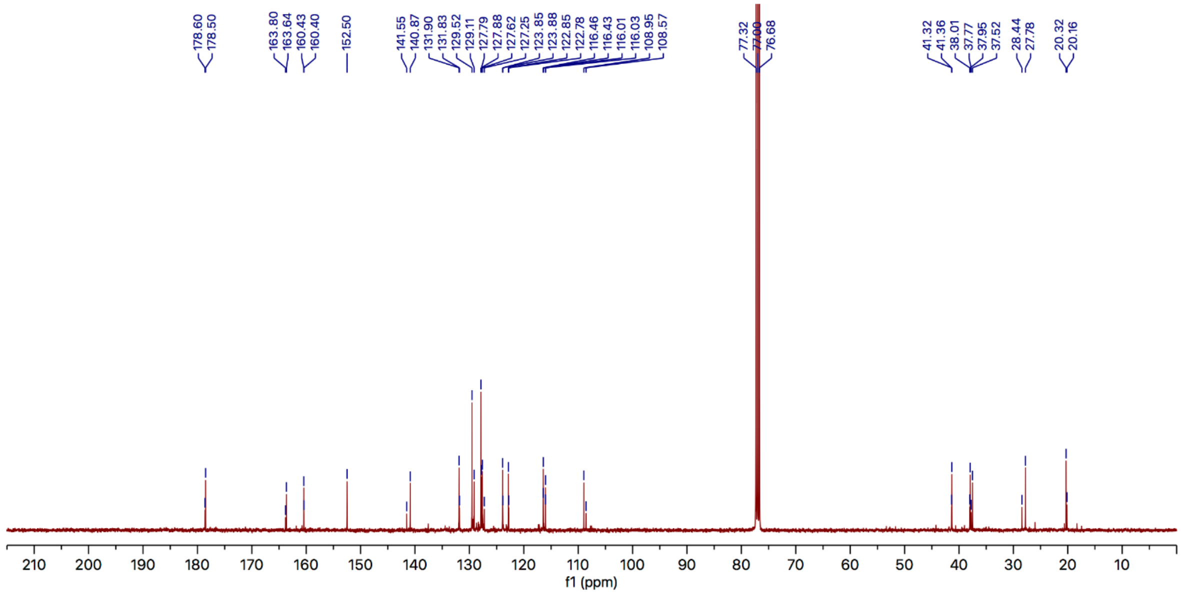

^{1}H (top) and ^{13}C (bottom) NMR spectra of compound **S5**

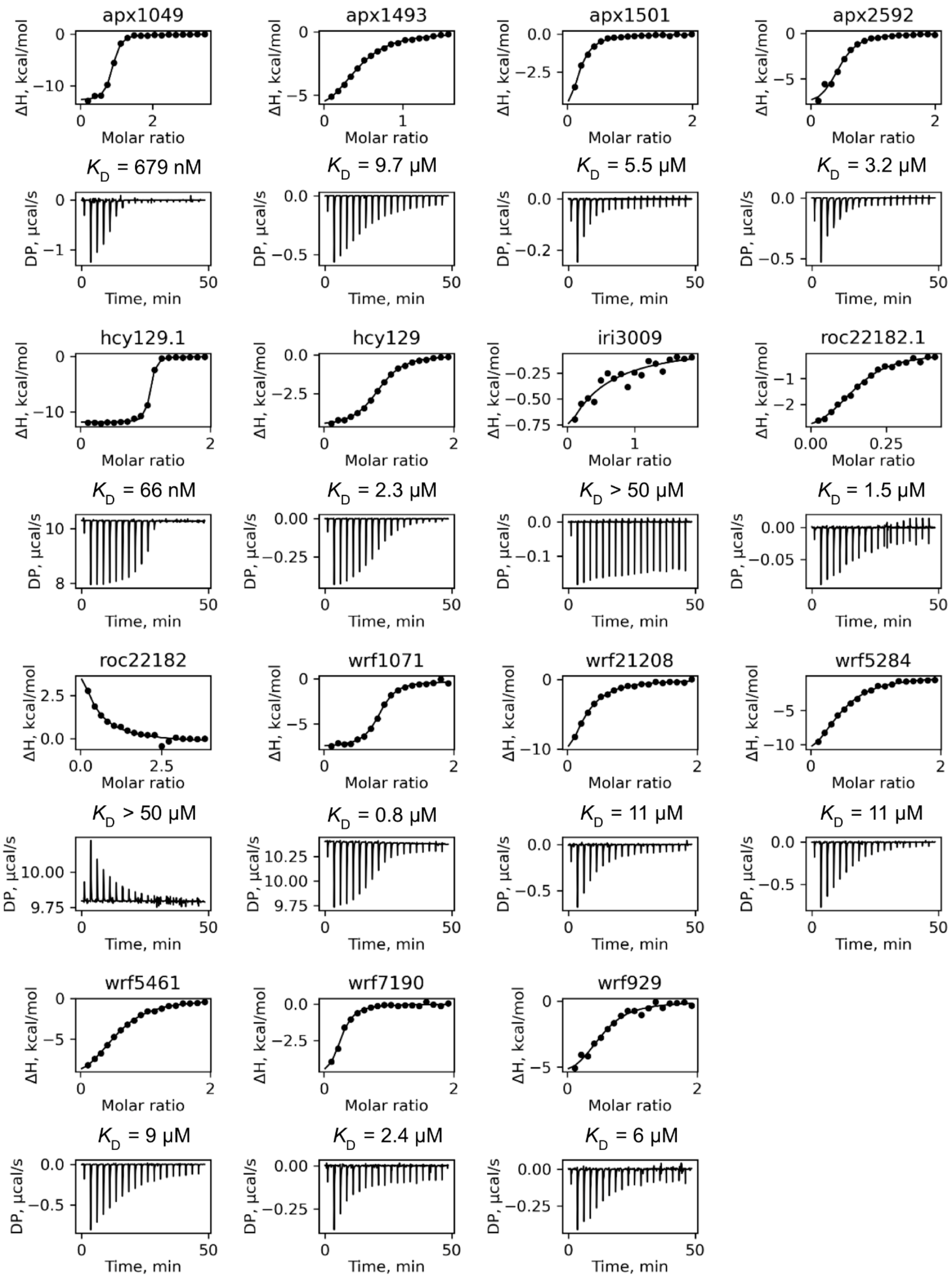

Supplementary Fig. 15. ITC binding isotherms. ITC binding isotherms and corresponding raw data for all characterized protein-ligand pairs.

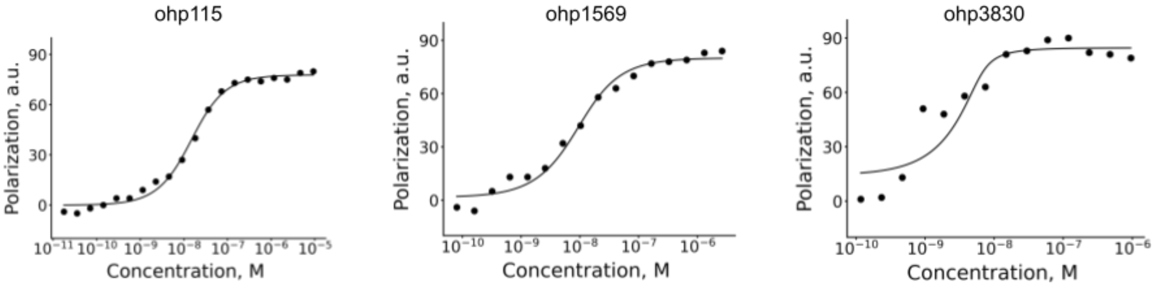

Supplementary Fig. 16. FP binding isotherm for OHP-binding proteins. OHP binding isotherms of designed proteins characterized using OHP-AF488.

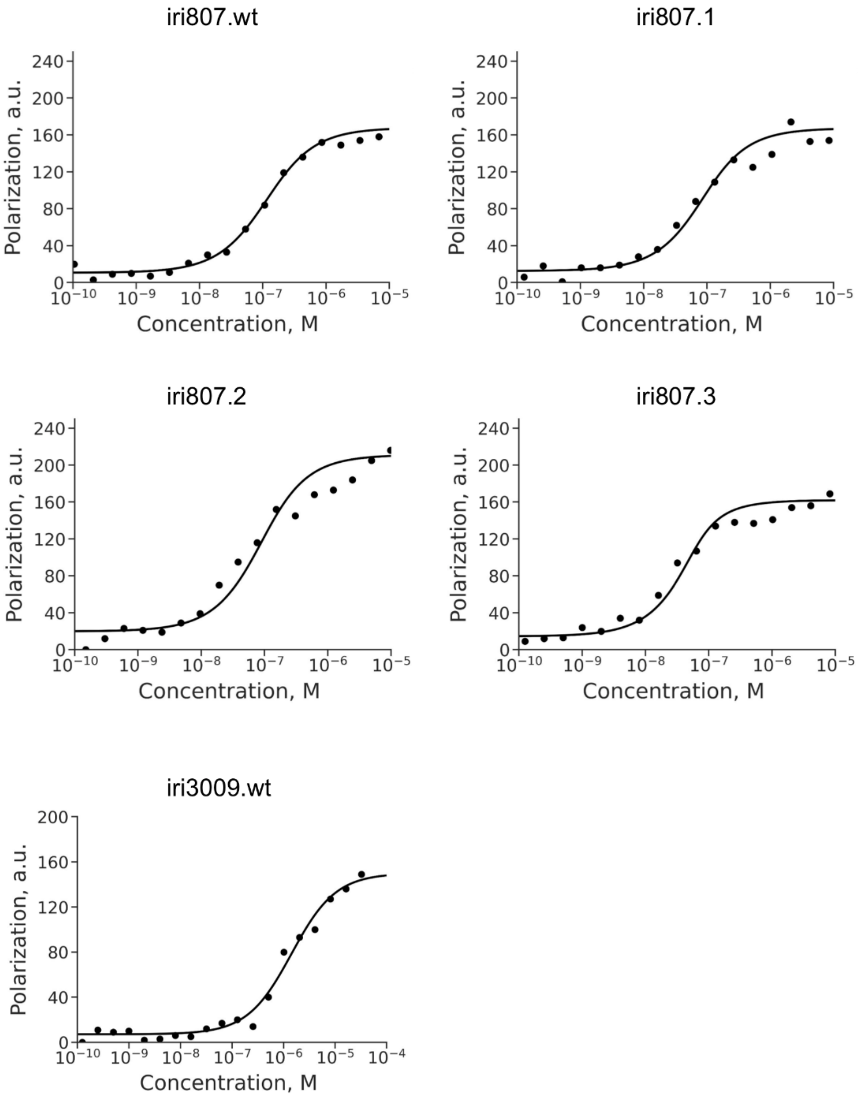

Supplementary Fig. 17. FP binding isotherm for SN-38-binding proteins. SN-38 binding isotherms of designed proteins characterized using SN-38-TAMRA.

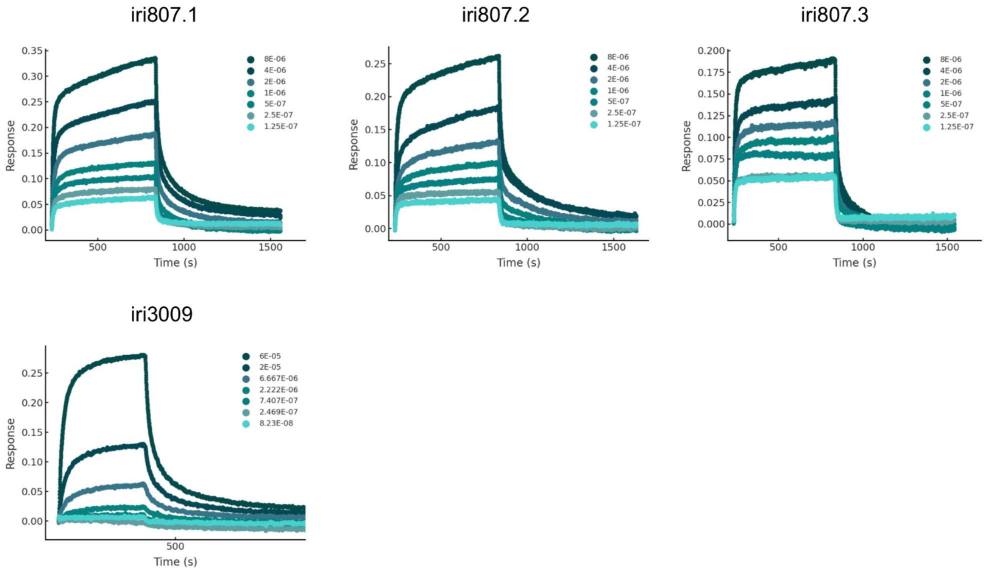

Supplementary Fig. 18. BLI titration sensograms for SN-38-binding proteins. BLI titration sensograms for designed SN-38-binding proteins characterized using biotin-SN-38.

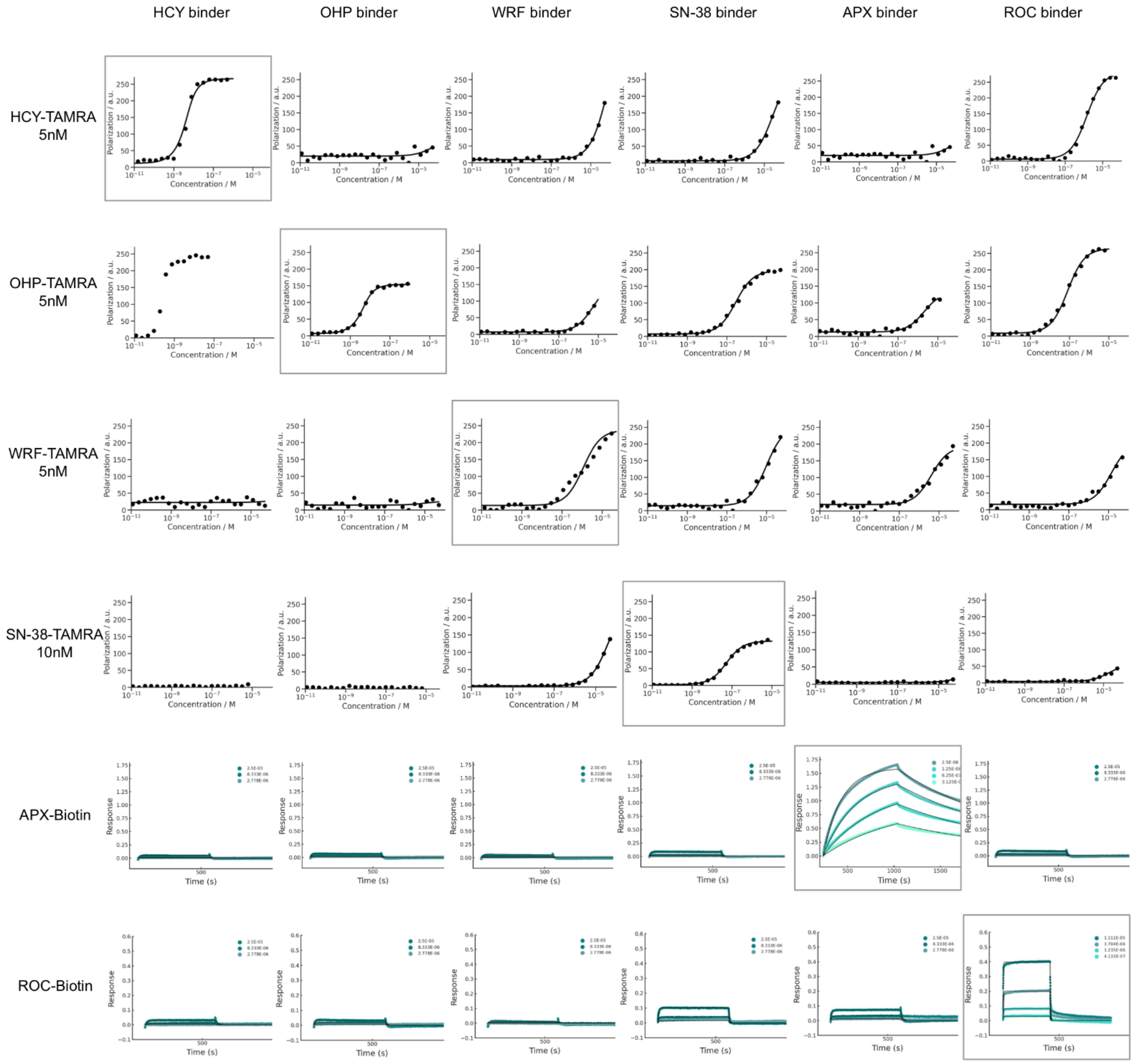

Supplementary Fig. 19. Binding traces of six representative binders against six small-molecule targets. HCY-, OHP-, WRF-, and SN-38-binding proteins were characterized by FP using TAMRA-labeled ligands, whereas APX- and ROC- binding proteins were characterized by BLI using biotin labeled ligands.

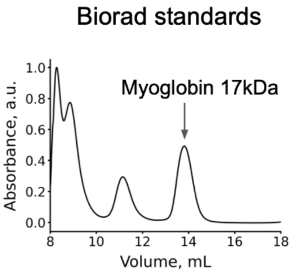

Supplementary Fig. 20. Size-exclusion chromatography profile of Bio-Rad protein standards. Elution profiles of Bio-Rad gel filtration standard proteins used for calibration.

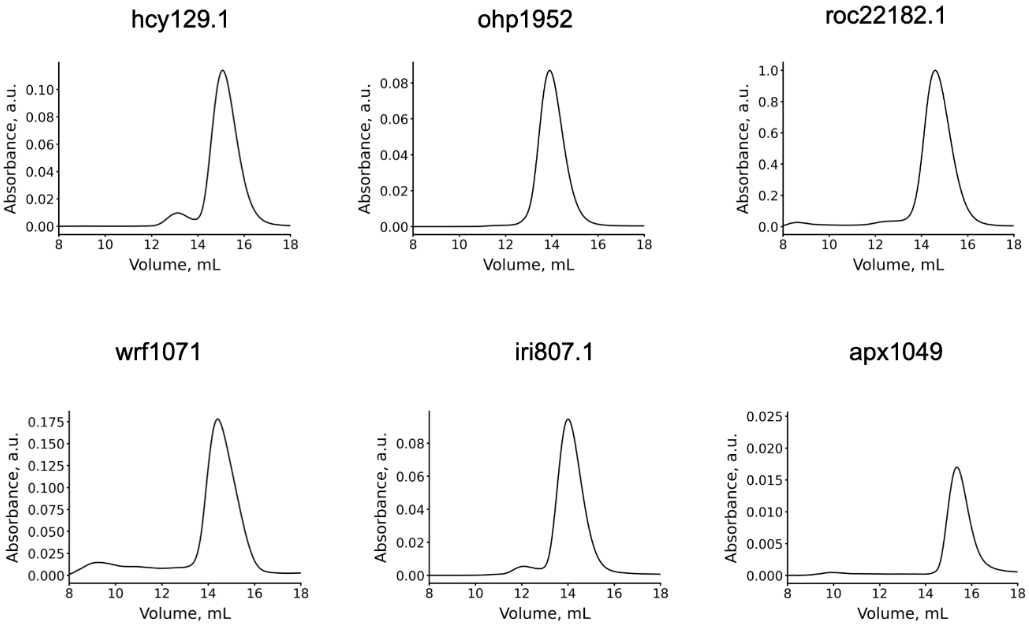

Supplementary Fig. 21. Size-exclusion chromatography profiles of six representative binders used for target specificity assessment. Elution profiles of purified representative binder proteins analyzed by size-exclusion chromatography.

Supplementary Table 1. Rosetta and AF2 metric cutoffs used to select designs

**Design approach**	**Target**	**Rosetta CMS**	**Rosetta ddG**	**Rosetta nHb**	**AF2 pIDDT**	**AF2 Cα-RMSD (Å)**	**AF2 SC-RMSD (Å)**
1	HCY	250	-0.09*CMS	1	not applied	not applied	not applied
1	WRF	270	-0.13*CMS	1	not applied	not applied	not applied
1	ROC	SC 0.65*	top 50%	1	not applied	not applied	not applied
1	APX	300	-0.02*CMS	1	not applied	not applied	not applied
1	SN-38	250	-0.03*CMS	1	not applied	not applied	not applied
2-round1	OHP	200	-35	1	80	1	2
2-resample	OHP	200	-30	1	80	1	1.5
2-round1	APX	250	-40	2	80	1	2
2-resample	APX	280	-45	1	80	1	2
2-round1	SN-38	230	-40	2	80	1	2
2-resample	SN-38	230	-45	1	85	1	2

*Used shape complementarity calculated by Rosetta instead of CMS

Supplementary Table 2. Small-molecule binding characterization results

**Target**	**Design name**	**Number of mutations compared to the original design**	**K_D (μM)**	**K_D determination method**
Cortisol	hcy129	0	2.3	ITC
Cortisol	hcy129.1	4	0.065	ITC
Cortisol	hcy129.1_CID	7	0.064	ITC
Coritsol	hcy129_mpnn5	9	3.4	ITC
Warfarin	wrf1071	0	0.83	ITC
Warfarin	wrf929	0	5.9	ITC
Rocuronium	roc22182	0	>50	ITC
Rocuronium	roc22182.1	9	>4	ITC
Apixaban	apx1049	0	0.679	ITC
Apixaban	apx1493	0	9.67	ITC
Apixaban	apx1501	0	5.5	ITC
Apixaban	apx2592	0	3.16	ITC
17α-hydroxy progesterone-AF488	ohp115	0	0.012	FP
17α-hydroxy progesterone-AF488	ohp3830	0	<0.006	FP
17α-hydroxy progesterone-AF488	ohp1569	0	0.006	FP
17α-hydroxy progesterone-AF488	ohp1952	0	0.017	FP
SN-38-TAMRA	iri807	0	0.090	FP
SN-38-Biotin	iri807	0	n/a	BLI
SN-38-TAMRA	iri807.1	3	0.059	FP
SN-38-Biotin	iri807.1	3	0.77	BLI (kinetic analysis*)

SN-38-Biotin	iri807.1	3	5.1	BLI (Binding isotherm fit with equilibrium response*)
SN-38-TAMRA	iri807.2	3	0.063	FP
SN-38-Biotin	iri807.2	3	1.0	BLI (kinetic analysis*)
SN-38-TAMRA	iri807.3	3	0.016	FP
SN-38-Biotin	iri807.3	3	5.47	BLI (kinetic analysis*)
SN-38-TAMRA	iri3009	0	1.41	FP
SN-38-Biotin	iri3009	0	39	BLI (kinetic analysis*)

Supplementary Table 3. Data collection and refinement statistics for the crystal structures of hcy129_mpnn5 and apx1049

	**hcy129_mpnn5 (PDB: 8UQF)**	**apx1049 (PDB: 8VFQ)**	**apx1049 (PDB: 8VEZ)**
**Resolution range**	47.9 - 1.52 (1.57- 1.52)	40.82 - 2.1 (2.31 - 2.1)	41.75 - 2.15 (2.37 - 2.15)
**Space group**	$P2_1$	$P\ 2_1 2_1 2_1$	$C\ 2\ 2\ 2_1$
**Unit cell**	48, 48, 56.4; 90, 94, 90	55.47, 60.27, 63.27, 90, 90, 90	54.97, 64.17, 60.10; 90,0 90, 90
**Unique reflections**	36533 (3628)	12166 (2950)	6020 (1474)
**Multiplicity**	2.5 (2.4)	9.1 (7.1)	9.8 (9.6)
**Completeness (%)**	90.3 (91.5)	94.43 (93.59)	99.70 (99.53)
**Mean I/sigma(I)**	11.7 (1.4)	5.7 (1.8)	6.67 (0.91)
**Wilson B-factor**	25.1	24.65	47.40
**R-merge**	0.06 (0.83)	0.911 (0.848	0.221 (2.419)
**R-pim**	0.04 (0.59)	0.096 (0.350)	0.075 (0.816)
**CC1/2**	0.99 (0.61)	0.990 (0.943)	0.996 (0.460)
**Reflections used in refinement**	36181 (3627)	12165 (2950)	6020 (1474)
**Reflections used for R-free**	1996 (195)	610 (144)	603 (148)
**R-work**	0.23 (0.34)	0.2735 (0.3093)	0.2229 (0.3280)
**R-free**	0.24 (0.35)	0.3232 (0.3471)	0.2774 (0.3627)
**Number of non-hydrogen atoms**	2155	1917	951
**macromolecules**	2022	1770	897
**ligands**	57	68	34
**solvent**	76	81	20
**Protein residues**	246	225	115
**RMS(bonds)**	0.024	0.003	0.002
**RMS(angles)**	1.81	0.500	0.450
**Ramachandran favored (%)**	99.57	96.83	98.23

**Ramachandran allowed (%)**	0.43	2.26	1.77
**Ramachandran outliers (%)**	0.00	0.00	0.00
**Average B-factor**	33.11	26.19	55.38
**macromolecules**	32.97	26.19	55.86
**ligands**	29.27	22.88	43.78
**solvent**	39.89	29.12	53.54

Statistics for the highest-resolution shell are shown in parentheses.

Supplementary Table 4. The closest and farthest native KSI structure for each characterized binder measured by TMscore. The designs structurally validated with crystallography structure determination are marked with *.

Binder name	Closest KSI structure	Closest TMscore	Farthest KSI structure	Farthest TMscore
apx1049*	3ov4_A	0.80	3owu_A	0.75
apx1493	3nhx_A	0.78	2inx_A	0.74
apx1501	1ogz_A	0.77	3owu_A	0.73
apx2592	1ohs_A	0.77	6ucy_A	0.73
hcy129*	1ohs_A	0.80	3cpo_A	0.77
iri3009	3ov4_A	0.84	3owu_A	0.78
iri807	3ov4_A	0.83	3owu_A	0.78
ohp115	3ov4_A	0.78	6ucy_A	0.74
ohp1569	3ov4_A	0.78	6ucy_A	0.73
ohp1952	3ov4_A	0.83	3owu_A	0.78
ohp3830	3ov4_A	0.82	3owu_A	0.78
roc22182	1ohs_A	0.80	3owu_A	0.75
wrf1071	5ugi_A	0.84	3vgn_A	0.78
wrf21208	3ov4_A	0.84	3vgn_A	0.77
wrf5284	3ov4_A	0.84	6ufs_A	0.78
wrf5461	3ov4_A	0.81	6ucy_A	0.76
wrf7190	3ov4_A	0.85	3owu_A	0.79
wrf929	5ugi_A	0.82	3vgn_A	0.75

Supplementary Table 5. Chemical similarity between the targets and the ligands bound in KSI structures in the PDB represented by tanimoto coefficients. The highest tanimoto coefficient for each target is highlighted in bold.

**Ligand 3 letter name**	**Ligand full name**	**PDB ID**	**Targets**					
			HCY	OHP	WAF	IRI	APX	ROC
5SD	Androstanedione	1ohs	0.32	0.36	0.09	0.03	0.06	0.14
6VW	Nandrolone	5kp4	**0.86**	**0.97**	0.09	0.05	0.11	0.21
AND	Epiandrosterone	1e3r	0.69	0.77	0.09	0.05	0.11	0.21
ASD	4-Androstene-3,17-dione	6ubq,6tzd,6ucy,3nhx	0.73	0.82	0.08	0.04	0.09	0.14
DNX	3,4-Dinitrophenol	6c1j	0.02	0.01	0.09	0.12	0.11	0.05
DXC	Deoxycholic acid	1e3v,6uae,6uad	0.44	0.50	0.09	0.06	0.08	**0.24**
EQU	Equilenin	3ows,5kp1,3owu,6u4i,1ogz,5ugi,3m8c,3myt,3ov4	0.17	0.18	**0.33**	**0.16**	0.17	0.17
ESR	5Alpha-Estran-3,17-Dione	1ohp	0.32	0.36	0.09	0.03	0.06	0.14
FFP	2,6-Difluorophenol	2inx	0.02	0.02	0.11	0.06	0.08	0.11
FNN	3-Fluoro-4-nitrophenol	3vgn	0.03	0.04	0.10	0.13	0.12	0.10
FP2	2-Fluorophenol	3cpo	0.02	0.02	0.11	0.06	0.07	0.10
IPH	Phenol	2pzv	0.02	0.02	0.12	0.05	0.06	0.05
J3Z	Estrone	7ry4	0.18	0.19	0.30	**0.16**	**0.19**	0.18
LLK	2-Acetyl-6-methoxynaphthalene	4cdl	0.15	0.16	0.23	0.13	0.15	0.14
Q6J	5Alpha-dihydronandrolone	6ufs	0.45	0.51	0.09	0.05	0.08	0.23

Supplementary Table 6. Binding affinities (K_D (μM)) of each of the six representative binders measured against all six targets. Raw binding traces can be found in Supplementary Information.

**Target used to measure binding affinity (K_D (μM))**	**Binder name and the target originally designed for**						
	hcy129.1 (HCY)	ohp1952 (OHP)	wrf1071 (WRF)	iri807.1 (IRI)	apx1049 (APX)	roc22182.1 (ROC)	Control protein: Albumin
HCY-TAMRA	**0.005**	No binding	67	26	No binding	1.4	No binding
OHP-TAMRA	0.005	**0.005**	No binding	0.27	No binding	0.078	52
WRF-TAMRA	No binding	No binding	**1.1**	9.7	4.0	No binding	5.0
SN-38-TAMRA	No binding	No binding	42	**0.048**	No binding	No binding	No binding
APX-Biotin	3556	39	1783	4381	**0.40**	3171	No binding
ROC-Biotin	3914	24	2563	75	43	**11**	No binding

Supplementary References

1. Wilbur, D. S. et al. Antibody Fragments in Tumor Pretargeting. Evaluation of Biotinylated Fab' Colocalization with Recombinant Streptavidin and Avidin. Bioconjug. Chem. 7, 689–702 (1996).
2. Dolence, E. K., Dolence, J. M., and Poulter, C. D., Solid-Phase Synthesis of a Radiolabeled, Biotinylated, and Farnesylated Ca(1)a(2)X Peptide Substrate for Ras- and a-Mating Factor Converting Enzyme. Bioconjug. Chem. 12, 35–43 (2001).
3. Mao, D. et al. A Synthetic Hybrid Molecule for the Selective Removal of Human Pluripotent Stem Cells from Cell Mixtures. Angew. Chem. Int. Ed. 56, 1765–1770 (2017).
4. Dou, J. et al. Sampling and energy evaluation challenges in ligand binding protein design. Protein Sci. 26, 2426–2437 (2017).
