# Supplementary material for: Small-molecule binding and sensing with a designed protein family
Source: Nat Commun. 2026 Mar 28;17:4533. doi: 10.1038/s41467-026-70953-8 (PMC13194776; doi:10.1038/s41467-026-70953-8)
Supplement: Supplementary file 2 — Reporting Summary [file 41467_2026_70953_MOESM2_ESM.pdf]

nature portfolio

Corresponding author(s): Gyu Rie Lee, Andy Hsien-Wei Yeh, and David Baker

Last updated by author(s): Dec 7, 2025

Reporting Summary

Nature Portfolio wishes to improve the reproducibility of the work that we publish. This form provides structure for consistency and transparency in reporting. For further information on Nature Portfolio policies, see our Editorial Policies and the Editorial Policy Checklist.

Statistics

For all statistical analyses, confirm that the following items are present in the figure legend, table legend, main text, or Methods section.

n/a	Confirmed	
☐	☒	The exact sample size (n) for each experimental group/condition, given as a discrete number and unit of measurement
☐	☒	A statement on whether measurements were taken from distinct samples or whether the same sample was measured repeatedly
☒	☐	The statistical test(s) used AND whether they are one- or two-sided<br>*Only common tests should be described solely by name; describe more complex techniques in the Methods section.*
☒	☐	A description of all covariates tested
☒	☐	A description of any assumptions or corrections, such as tests of normality and adjustment for multiple comparisons
☒	☐	A full description of the statistical parameters including central tendency (e.g. means) or other basic estimates (e.g. regression coefficient) AND variation (e.g. standard deviation) or associated estimates of uncertainty (e.g. confidence intervals)
☒	☐	For null hypothesis testing, the test statistic (e.g. F, t, r) with confidence intervals, effect sizes, degrees of freedom and P value noted<br>*Give P values as exact values whenever suitable.*
☒	☐	For Bayesian analysis, information on the choice of priors and Markov chain Monte Carlo settings
☒	☐	For hierarchical and complex designs, identification of the appropriate level for tests and full reporting of outcomes
☒	☐	Estimates of effect sizes (e.g. Cohen's d, Pearson's r), indicating how they were calculated

Our web collection on statistics for biologists contains articles on many of the points above.

Software and code

Policy information about availability of computer code

Data collection	The computational design scripts were written based on PyRosetta, Rosetta, and LigandMPNN. The design scripts developed for the work are uploaded on Zenodo and can be publicly accessed at https://doi.org/10.5281/zenodo.17847477.<br><br>The Rosetta macromolecular modeling and design software package (https://www.rosettacommons.org) is freely available to academic and non-commercial users. Commercial licenses can be requested through the University of Washington Technology Transfer Office. The source code of RIFDock is freely available at https://github.com/rifdock/rifdock.<br>PyRosetta is a Python-based interface to the Rosetta modeling suite (https://www.pyrosetta.org). Both free Academic and Commercial licenses are available.<br>Source code of the LigandMPNN model that was used for sequence design is available under an open-source license at https://github.com/dauparas/LigandMPNN.
Data analysis	For data analysis and visualization we used Python v3.9.6 (https://www.python.org/), Seaborn v0.11.2, Matplotlib v3.5.3 (https://github.com/matplotlib/matplotlib), NumPy v1.24.1 (https://github.com/numpy/numpy), and pandas v1.4.3 (https://pandas.pydata.org/). Protein structure figures were created with Pymol (https://github.com/schrodinger/pymol-open-source). The software PEAR (https://cme.h-its.org/exelixis/web/software/pear) was used for NGS sequence analysis, and dnaworks (https://helixweb.nih.gov/dnaworks/) was used for DNA oligo sequence optimization. FlowJo was used for flow cytometry data analysis.

For manuscripts utilizing custom algorithms or software that are central to the research but not yet described in published literature, software must be made available to editors and reviewers. We strongly encourage code deposition in a community repository (e.g. GitHub). See the Nature Portfolio guidelines for submitting code & software for further information.

Data

Policy information about availability of data

All manuscripts must include a data availability statement. This statement should provide the following information, where applicable:

- Accession codes, unique identifiers, or web links for publicly available datasets
- A description of any restrictions on data availability
- For clinical datasets or third party data, please ensure that the statement adheres to our policy

Crystal structures of the cortisol binding protein and apixaban binding protein are available in the PDB database (https://www.rcsb.org/) with the accession codes 8UQF, 8VEZ, and 8VFQ. The deep sequencing data have been deposited in the NCBI database with the accession ID PRJNA1356499 [https://www.ncbi.nlm.nih.gov/bioproject/PRJNA1356499].

Research involving human participants, their data, or biological material

Policy information about studies with human participants or human data. See also policy information about sex, gender (identity/presentation), and sexual orientation and race, ethnicity and racism.

Reporting on sex and gender	N/A
Reporting on race, ethnicity, or other socially relevant groupings	N/A
Population characteristics	N/A
Recruitment	N/A
Ethics oversight	N/A

Note that full information on the approval of the study protocol must also be provided in the manuscript.

Field-specific reporting

Please select the one below that is the best fit for your research. If you are not sure, read the appropriate sections before making your selection.

☒ Life sciences ☐ Behavioural & social sciences ☐ Ecological, evolutionary & environmental sciences

For a reference copy of the document with all sections, see nature.com/documents/nr-reporting-summary-flat.pdf

Life sciences study design

All studies must disclose on these points even when the disclosure is negative.

Sample size	The sample sizes were not predetermined with any statistical method.
Data exclusions	No sample was excluded from analysis
Replication	Experimental binding assays using the methods ITC, FP, and BLI were repeated at least twice with a different batch of protein sample, and resulted in similar results.
Randomization	For yeast cell library sorting, the cells were randomly selected and separated upon performing cell sorting.
Blinding	N/A (Does not apply to the experiments carried out in this study)

Reporting for specific materials, systems and methods

We require information from authors about some types of materials, experimental systems and methods used in many studies. Here, indicate whether each material, system or method listed is relevant to your study. If you are not sure if a list item applies to your research, read the appropriate section before selecting a response.

Materials & experimental systems

n/a	Involved in the study	
	☒	Antibodies
☒	☐	Eukaryotic cell lines
☒	☐	Palaeontology and archaeology
☒	☐	Animals and other organisms
☒	☐	Clinical data
☒	☐	Dual use research of concern
☒	☐	Plants

Methods

n/a	Involved in the study	
☒	☐	ChIP-seq
☐	☒	Flow cytometry
☒	☐	MRI-based neuroimaging

Antibodies

Antibodies used	anti-cMyc tag antibody conjugated to FITC
Validation	The antibody was validated and provided by the vendor (Immunology Consultants Laboratory) https://www.icllab.com/anti-c-myc-antibody-chicken-fitc-conjugated-cmyc-45f.html

Plants

Seed stocks	N/A
Novel plant genotypes	N/A
Authentication	N/A

Flow Cytometry

Plots

Confirm that:

☒ The axis labels state the marker and fluorochrome used (e.g. CD4-FITC).

☒ The axis scales are clearly visible. Include numbers along axes only for bottom left plot of group (a 'group' is an analysis of identical markers).

☒ All plots are contour plots with outliers or pseudocolor plots.

☒ A numerical value for number of cells or percentage (with statistics) is provided.

Methodology

Sample preparation	EBY100 strain yeast cells and linearized pETCON3 vector were used to transform the design oligonucleotide library with recombination.
Instrument	SONY SH800 Cell Sorter was used for fluorescence activated cell sorting.<br>Attune NxT Flow Cytometer (Thermo Fisher) was used to analyze individual clones.
Software	The provided softwares of the SONY cell sorter and the Attune NxT Flow Cytometer were used to collect data, and we used FlowJo for data analysis and visualization.
Cell population abundance	At least 1 million yeast cells were randomly pulled from the culture upon cell sorting, and we collected from few hundreds to millions of cells depending on the collection fraction.
Gating strategy	We applied gates to exclude outliers using FSC-A/SSC-A, followed by a gating strategy using FSC-A/FSC-H to exclude doublets. For the activity (binding) sort we used the negative control samples to set gates.

☒ Tick this box to confirm that a figure exemplifying the gating strategy is provided in the Supplementary Information.
